# Lrg1 Regulates β (1,3)-Glucan Masking in Candida albicans through the Cek1 MAP Kinase Pathway

**DOI:** 10.1128/mBio.01767-19

**Published:** 2019-09-17

**Authors:** Tian Chen, Andrew S. Wagner, Robert N. Tams, James E. Eyer, Sarah J. Kauffman, Eric R. Gann, Elias J. Fernandez, Todd B. Reynolds

**Affiliations:** a Department of Microbiology, The University of Tennessee, Knoxville, Tennessee, USA; b Department of Biochemistry & Cellular and Molecular Biology, The University of Tennessee, Knoxville, Tennessee, USA; Duke University

**Keywords:** GTPase, Cdc42, Rho1, Ras1, Rac1, MAPK cascade, Ste11, Cek1, cell wall, β (1,3)-glucan, virulence, *Candida albicans*

## Abstract

Candida albicans is among the most prevalent opportunistic human fungal pathogens. The ability to mask the immunogenic polysaccharide β (1,3)-glucan from immune detection via a layer of mannosylated proteins is a key virulence factor of C. albicans. We previously reported that hyperactivation of the Cek1 mitogen-activated protein (MAP) kinase pathway promotes β (1,3)-glucan exposure. In this communication, we report a novel upstream regulator of Cek1 activation and characterize the impact of Cek1 activity on fungal virulence. Lrg1 encodes a GTPase-activating protein (GAP) that has been suggested to inhibit the GTPase Rho1. We found that disruption of *LRG1* causes Cek1 hyperactivation and β (1,3)-glucan unmasking. However, when GTPase activation was measured for a panel of GTPases, the *lrg1ΔΔ* mutant exhibited increased activation of Cdc42 and Ras1 but not Rho1 or Rac1. Unmasking and Cek1 activation in the *lrg1ΔΔ* mutant can be blocked by inhibition of the Ste11 MAP kinase kinase kinase (MAPKKK), indicating that the *lrg1ΔΔ* mutant acts through the canonical Cek1 MAP kinase cascade. In order to determine how Cek1 hyperactivation specifically impacts virulence, a doxycycline-repressible hyperactive *STE11^ΔN467^* allele was expressed in C. albicans. In the absence of doxycycline, this allele overexpressed *STE11^ΔN467^*, which induced production of proinflammatory tumor necrosis factor alpha (TNF-α) from murine macrophages. This *in vitro* phenotype correlates with decreased colonization and virulence in a mouse model of systemic infection. The mechanism by which Ste11^ΔN467^ causes unmasking was explored with RNA sequencing (RNA-Seq) analysis. Overexpression of Ste11^ΔN467^ caused upregulation of the Cph1 transcription factor and of a group of cell wall-modifying proteins which are predicted to impact cell wall architecture.

## Abstract (precis)

Candida albicans is an important source of systemic infections in humans. The ability to mask the immunogenic cell wall polymer β (1,3)-glucan from host immune surveillance contributes to fungal virulence. We previously reported that the hyperactivation of the Cek1 MAP kinase cascade promotes cell wall unmasking, thus increasing strain immunogenicity. In this study, we identified a novel regulator of the Cek1 pathway called Lrg1. Lrg1 is a predicted GTPase-activating protein (GAP) that represses Cek1 activity by downregulating the GTPase Cdc42 and its downstream MAPKKK, Ste11. Upregulation of Cek1 activity diminished fungal virulence in the mouse model of infection, and this correlates with increased cytokine responses from macrophages. We also analyzed the transcriptional profile determined during β (1,3)-glucan exposure driven by Cek1 hyperactivation. Our report provides a model where Cek1 hyperactivation causes β (1,3)-glucan exposure by upregulation of cell wall proteins and leads to more robust immune detection *in vivo*, promoting more effective clearance.

## INTRODUCTION

*Candida* species are the fourth most common cause of bloodstream infections in hospitalized patients in the United States (1). Despite the availability of several effective antifungals, the mortality rate of these infections still exceeds 40% (1). Current antifungal drugs for treatment of *Candida* infections include polyenes, azoles, and echinocandins; however, mortality rates are unacceptably high even after accounting for limitations such as drug resistance or toxicity. This suggests that other therapeutic approaches need to be used in conjunction with anti-infectives. One approach is to improve the residual immune response of patients to these pathogens (2–4). The immune response could be enhanced by making fungal pathogens more visible to the immune system’s sensory cells. The fungal cell wall is a major focus for this as it is the main interface between the immune system and the fungus (5).

The C. albicans cell wall is composed of two major layers, where the outer layer is enriched for glycosylated proteins (mannan), and the inner layer consists of chitin, β (1,6)-glucan, and the highly immunogenic β (1,3)-glucan. Fungal pathogens have a diversity of mechanisms to manipulate cell wall architecture to mask proinflammatory β (1,3)-glucan epitopes from host recognition. In Histoplasma capsulatum, α-glucan serves a masking function by concealing the β-glucan, while the Eng1 exoglucanase hydrolyzes β-(1,3)-glycosyl bonds and removes exposed β-glucans not covered by α–glucan (6, 7). Aspergillus fumigatus Uge3 regulates the biosynthesis of galactosaminogalactan, a polymer that protects hyphal β-glucan from immune detection (8). In C. albicans, β (1,3)-glucan is masked by the outer layer of mannan. Certain genetic mutations, treatment with the cell wall inhibitor caspofungin, or damage by neutrophils can expose C. albicans β (1,3)-glucan (9–11). This exposure facilitates recognition by host immune cells through receptors such as Dectin-1 and therefore launches immune responses more efficiently and rapidly, including induction of proinflammatory cytokines such as tumor necrosis factor alpha (TNF-α), which promotes fungal clearance (12). The importance of β (1,3)-glucan exposure and the resultant enhancing of immune detection are becoming better appreciated in medical mycology, and could lead to discoveries that will improve adjunctive therapy approaches (9, 11, 13–16).

We reported previously that the hyperactivated GTPase mutation *RHO1^Q67L^* causes β (1,3)-glucan unmasking in C. albicans (17). The most well-known contribution of Rho1 in this organism is that of maintaining cell wall architecture by regulating several downstream effectors, including the β(1,3)-glucan synthase catalytic subunit Fks1, the protein kinase C homolog Pkc1, and the downstream cell wall integrity mitogen-activated protein kinase (MAPK) cascade containing Mkc1 (18–21). Rho1 serves as a molecular switch that cycles between an active GTP-bound state and an inactive GDP-bound state (22). Two main types of regulatory proteins control the GTP/GDP state of GTPases. These include guanine nucleotide exchange factors (GEFs), which stimulate GTP to be loaded onto the enzyme and keep it in the active GTP-bound state, and GTPase-activating proteins (GAPs), which promote the hydrolysis of GTP and convert the enzyme into the inactive GDP-bound state (23). In C. albicans, Lrg1 is proposed to act as the Rho1 GAP based on the evidence that the *lrg1ΔΔ* mutant and hyperactivated *RHO1^Q67L^* mutant each induce hyphal formation, and a mutation of the PKC1 gene, which acts downstream of Rho1, blocks *lrg1*∆∆ hyperfilamentation. These data suggest that Lrg1 could negatively regulate Rho1 activity (24, 25), but the GAP activity of Lrg1 for Rho1 has not been measured in C. albicans (24).

Like Rho1, Cdc42 is an essential GTPase in C. albicans. Cdc42 is required for cell viability, polarized growth, and yeast-to-hypha morphogenesis (26–28). Like that of other GTPases, Cdc42 activity is regulated by GAPs negatively and GEFs positively (29, 30). Activated Cdc42 turns on downstream effectors, including p21-activated kinase (PAK) Cst20, which further initiates signaling through the Cek1 MAPK cascade (17, 26). The Cek1 MAPK cascade, comprised of Ste11-Hst7-Cek1, is primarily responsible for gene transcription involved in morphogenesis, cell wall stress adaption, and cell growth (17, 31–34). In C. albicans, Cek1 has been reported to respond to several GTPases, including Cdc42, Rho1, Ras1, and Rac1, although most of these data are genetic in nature (17, 35–37). Here, we demonstrate for the first time that in C. albicans, the Lrg1 GAP protein negatively controls Cdc42 and Ras1 activity *in vivo* but not the activity of Rho1 or Rac1. Moreover, Cek1 is activated downstream of a *lrg1*ΔΔ mutant by a pathway(s) that utilizes the canonical Ste11 MAP kinase kinase kinase (MAPKKK). Furthermore, we reveal that hyperactivation of Cek1 by a *STE11^ΔN467^* allele compromises fungal virulence in the mouse model of systemic infection. Finally, we show that upregulated Cek1 likely activates unmasking through the activity of the downstream transcription factor Cph1 and some of its cell wall-related transcriptional targets.

## RESULTS

### The *lrg1ΔΔ* mutation causes β (1,3)-glucan exposure in C. albicans.

**FIG 1:**
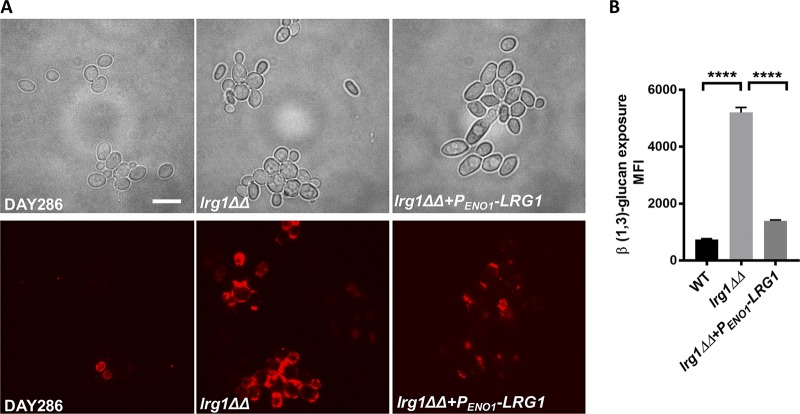
Lrg1 represses β (1,3)-glucan unmasking in C. albicans. (A) Cells were cultured overnight in YPD medium and stained with anti-β (1,3)-glucan antibody and Cy3-conjugated secondary antibody. Cells were imaged by epifluorescence microscopy. The scale bar represents 10 μm. (B) Cells were stained with anti-β (1,3)-glucan antibody and phycoerythrin (PE)–conjugated secondary antibody. Flow cytometry was performed to quantify β (1,3)-glucan exposure in different *Candida* cells. The statistical analysis was performed using one-way ANOVA. ****, *P* < 0.0001. MFI, mean fluorescent intensity; WT, wild type.

Lrg1 homologs impact several cell wall-related functions in fungi (e.g., cell wall integrity, cell fusion, and morphogenesis) (24, 25, 38, 39). In S. cerevisiae, Lrg1 stimulates the intrinsic GTPase activity of Rho1 and therefore converts Rho1 to its inactive, GDP-bound state (25, 38). We previously showed that hyperactive Rho1 (Rho1^Q67L^) exposed β (1,3)-glucan in the cell wall but appeared to act through the Cek1 MAPK rather than Mkc1 (17). Since Lrg1 has been described as the Rho1 GAP in C. albicans, we hypothesized that disruption of *LRG1* would cause β (1,3)-glucan exposure. To this end, we performed immunofluorescence staining with anti-β (1,3)-glucan antibody (Ab) on a *lrg1ΔΔ* mutant (40). As shown in Fig. 1A, β (1,3)-glucan is noticeably more exposed in *lrg1ΔΔ* cells than in wild-type cells or the complemented strain, where *LRG1* expression is under the regulation of constitutive promoter *P_ENO1_*. Flow cytometry confirmed that the *lrg1ΔΔ* mutant has significantly increased levels of β (1,3)-glucan unmasking compared to control strains (Fig. 1B). This reveals that Lrg1 acts as a repressor of cell wall β (1,3)-glucan unmasking in C. albicans.

### *LRG1* disruption upregulates the Cek1 MAPK activity.

**FIG 2:**
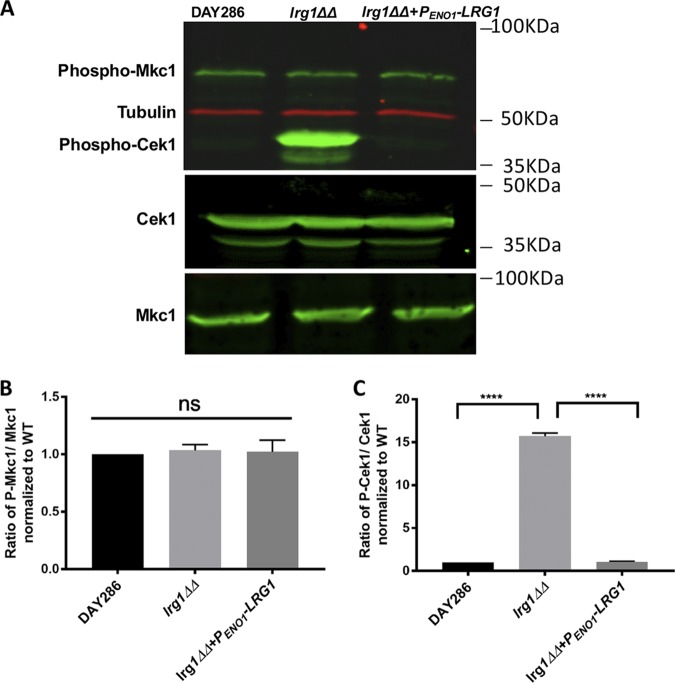
The Cek1 MAPK is hyperphosphorylated in *lrg1ΔΔ* cells compared to wild type. (A) Proteins were isolated from *Candida* cells in log phase. Western blotting was performed with anti-phospho-p44/42 antibody (stains phosphorylated Mkc1 and Cek1), as well as anti-Mkc1 and-Cek1 antibodies (stain total Mkc1 and Cek1 proteins, respectively). (B) The phospho-Mkc1 bands were quantified and normalized based on the total Mkc1 bands and tubulin. (C) The phospho-Cek1 bands were quantified and normalized based on the total Cek1 bands and tubulin. The graphs in both cases were based on quantification of 3 blots, and statistical analysis was performed using one-way ANOVA. ****, *P* < 0.0001; ns, not significant.

Several MAPK pathways are involved in the regulation of cell wall architecture in C. albicans (31, 34, 41), and improper activation of the Cek1 MAPK by upstream GTPases such as Cdc42 or Rho1 causes β(1,3)-glucan exposure (17). Lrg1 is a proposed Rho1 GAP in C. albicans (24) and should upregulate Mkc1 and possibly Cek1. To evaluate this, we performed Western blotting to detect the activated (phosphorylated) forms of Cek1 and Mkc1 in C. albicans. As shown in Fig. 2, the *lrg1ΔΔ* mutant did not visibly increase the intensity of phosphorylated Mkc1; instead, Cek1 was hyperphosphorylated at a level up to 15-fold higher than that seen with the wild type. This indicates that Lrg1 represses the activity of Cek1 instead of Mkc1 in C. albicans and that this might be responsible for the cell wall exposure in the *lrg1ΔΔ* mutant.

### Disruption of the *LRG1* gene hyperactivates the small GTPases Cdc42 and Ras1.

**FIG 3:**
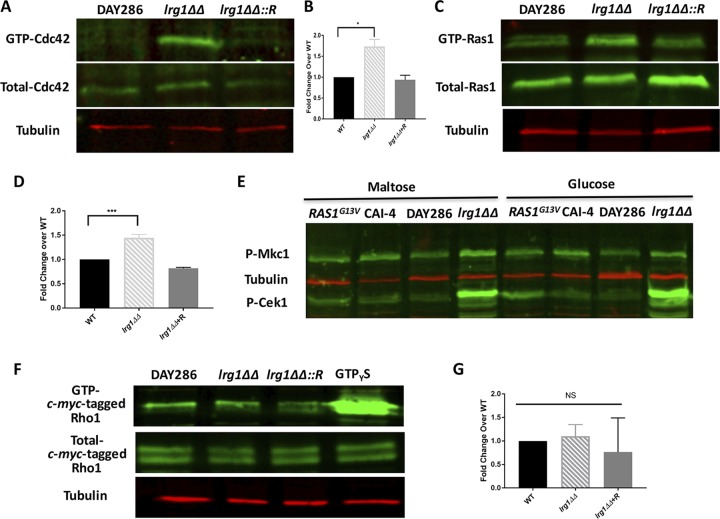
Lrg1 inhibits the activity of GTPases Cdc42 and Ras1, but not Rho1. (A) GTP-Cdc42 was pulled down by glutathione beads conjugated with glutathione *S*-transferase (GST)-PAK1, which specifically binds active GTP-Cdc42/Rac1. Western blotting was performed on the isolated GTP-Cdc42 portion with anti-Cdc42 antibody, and tubulin was used as a loading control. (B) The ratio of GTP-bound Cdc42/Total-Cdc42 to the WT level was calculated. The densitometries of the active GTP-Cdc42 band and total Cdc42 band were quantified by Image J based on 2 blots. *, *P* = 0.0261. (C) Active GTP-Ras1 was pulled down by using the Ras1 binding domain (RBD) within Raf1 as a probe, followed by Western blotting using anti-Ras1 antibody to evaluate the amount of active Ras1. (D) The ratio of GTP-bound Ras1/Total-Ras1 to the WT level was calculated as described for panel B. ***, *P* = 0.0006. (E) Western blotting was performed with anti-phospho-p44/42 antibody to assess the phosphorylation of Cek1 in the hyperactive *RAS1^G13V^* mutant. The *RAS1^G13V^* mutant was in the CAI-4 background, and the allele was expressed from the maltose promoter *P_MAL_*, so strains were compared in media with maltose or glucose. (F) The active GTP-bound c-*myc*-tagged Rho1 was isolated by incubating with the purified GST-RID protein. The mixture was incubated with glutathione beads. Western blotting was performed with anti-c-*myc* antibody to measure the amount of GTP-bound Rho1 in the pulldowns. Total Rho1 was determined by performing Western blotting on the total protein extract. (G) The ratio of GTP-bound Rho1/Total-Rho1 to the WT levels was calculated as described for panel B. NS, no significance. *lrg1ΔΔ*::*R* or *lrg1ΔΔ+R* = reintegrant strain.

The small GTPase Cdc42 sits upstream of Cek1 and is essential for many cellular functions, including cellular polarized growth and bud emergence in C. albicans (26, 37, 42). Regulation of the GTP/GDP-binding state controls the Cdc42 activation state (26, 27). Given that Cdc42 is known to control Cek1 MAPK activity in C. albicans, we hypothesized that *LRG1* disruption activates Cek1 through Cdc42. To test this, we measured Cdc42 activity by quantifying the amount of GTP-bound active Cdc42 *in vivo*. As shown in Fig. 3A and B, the concentration of GTP-bound Cdc42 was clearly upregulated compared to that of wild-type DAY286 and the reintegrated strain. Thus, this suggests that Lrg1 controls Cdc42 activity negatively in C. albicans. Cdc42 has been shown to control Cek1 phosphorylation in this organism (17), so Cek1 hyperactivation in the *lrg1ΔΔ* mutant may be mediated by Cdc42.

There is a possibility that Lrg1 impacts other Cek1-associated GTPases. To address this, we measured the activity of the GTPase Rac1, which has been suggested to function upstream of Cek1 (36). Using a strain expressing Rac1 with a green fluorescence protein (GFP) tag, Rac1 activity was assessed by pulling down the GTP-bound GFP-Rac1. The Cdc42/Rac1 interactive binding (CRIB) protein used in the Cdc42 pulldown assay was used as the probe to isolate GTP-bound GFP-Rac1, while GFP antibody was utilized to detect the amount of GFP-Rac1 specifically. As shown in Fig. S1 in the supplemental material, the level of GTP-bound GFP-Rac1 in the *lrg1ΔΔ* mutant is reduced compared to that of wild type. This suggests that Rac1 and Cdc42 might work antagonistically when Lrg1 is disrupted in C. albicans.

10.1128/mBio.01767-19.2 FIG S1 Loss of Lrg1 decreases GTPase Rac1 activity. (A) Active GTP-bound GFP-tagged Rac1 was pulled down using GST-CRIB as a probe, followed by Western blotting with anti-GFP antibody. The amount of total Rac1 was also detected from whole-cell lysates by using anti-GFP antibody. (B) The GFP-Rac1 bands in the pulldown lanes were quantified by Image J and normalized to the total amount of GFP-Rac1. The data represent 2 blots. *, *P* = 0.0154. Download FIG S1, TIF file, 0.2 MB. Copyright © 2019 Chen et al. 2019 Chen et al. This content is distributed under the terms of the Creative Commons Attribution 4.0 International license.

Ras1 is an important GTPase for filamentation and controls the cyclic AMP signaling cascade and protein kinase A. It has been suggested to act upstream of Cek1 in C. albicans (35). Since Cek1 is hyperactive in the *lrg1ΔΔ* mutant (Fig. 2), it is reasonable to evaluate whether Ras1 activity would be upregulated when the *LRG1* gene was disrupted. We performed a pulldown assay to isolate active GTP-Ras1 by using its downstream effector Raf1 as a probe. The ratio of GTP-Ras1 to total Ras1 was significantly higher in the *lrg1ΔΔ* mutant than in control strains, indicating that *LRG1* disruption induced Ras1 activity albeit modestly, which is similar to results from Xie et al. (24) (Fig. 3C and D). To biochemically test if Ras1 can cause Cek1 hyperactivation, we performed Western blotting on a *P_MAL_*-*RAS1^G13V^* mutant (43) (hyperactive allele) with the phospho-p44/42 antibody that recognizes phosphorylated Cek1 and Mkc1. Unexpectedly, hyperactivated Ras1^G13V^ (expressed from the maltose promoter *P_MAL_*) did not induce Cek1 activation (Fig. 3E). Similar results were seen when the *RAS1^G13V^* allele was expressed from a constitutive *P_ENO1_* promoter (Fig. S2). These results indicate that Ras1 does not activate Cek1 downstream of the *lrg1ΔΔ* mutation in C. albicans.

10.1128/mBio.01767-19.3 FIG S2 Hyperactivated Ras1^G13V^ does not cause Cek1 phosphorylation. The *RAS1^G13V^* mutant allele was placed under regulation by the constitutive enolase promoter (*P_ENO1_*) and was transformed into the DAY286 background. Strains were cultured overnight in YPD medium at 30°C, diluted to an OD_600_ of 0.2, and grown for 3 h. Cells were collected, and Western blotting was performed with anti-P-p44/42 antibody to assess Cek1 phosphorylation. “1#” and “2#” indicate two transformants. Download FIG S2, TIF file, 0.5 MB. Copyright © 2019 Chen et al. 2019 Chen et al. This content is distributed under the terms of the Creative Commons Attribution 4.0 International license.

### Lrg1 does not act as the Rho1 GAP in C. albicans.

In S. cerevisiae, Lrg1 represses Rho1, and this has been supported by genetic data in C. albicans; therefore, we measured Rho1 activity in the *lrg1ΔΔ* mutant. Due to lack of a commercial reagent to pull down active GTP-Rho1, we expressed and purified a glutathione *S*-transferase (GST)-tagged Rho1 interactive domain (RID) protein specific for C. albicans GTP-Rho1 (44). In addition, CaRho1 was tagged with the c-*myc* epitope for Western blotting due to lack of available commercial CaRho1 antibody (45). However, as shown in Fig. 3F and G, the *lrg1ΔΔ* mutant did not contain a higher concentration of GTP-Rho1 than the wild type. The last lane in Fig. 3F includes wild-type extract incubated with GTPγS, which is a nonhydrolyzable substrate for GTPases whose use results in a strong signal and which acts as a positive control for GTP-*myc*-Rho1. The lack of an induction of Rho1 activity in the *lrg1ΔΔ* mutant suggests that Lrg1 does not act as the Rho1 GAP in C. albicans.

### Inhibition of Ste11 signaling blocks Cek1 hyperphosphorylation and β (1,3)-glucan exposure in the *lrg1*ΔΔ mutant.

**FIG 4:**
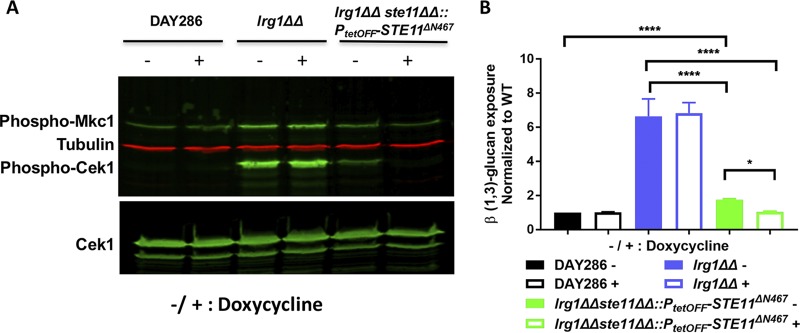
Disruption of *LRG1* causes increased Cek1 activation through the Ste11 MAPKKK. (A) One *STE11* allele was deleted in the *lrg1ΔΔ* mutant using the well-established SAT1-flipper method. The second *STE11* allele was replaced by the *P_tetOFF_-STE11^ΔN467^* allele through homologous recombination. The *Candida* cells were treated with 0.5 μg/ml of doxycycline overnight at 30°C, followed by subculturing in fresh YPD medium for 3 h at 30°C and growth to the log phase with or without doxycycline. Western blotting was performed with anti-phospho-p44/42 antibody for phosphorylated Cek1 and Mkc1. Anti-tubulin antibody was used to detect tubulin, and anti-Cek1 antibody was used to detect total Cek1. (B) The *Candida* cells were cultured overnight with or without doxycycline, followed by immunofluorescent staining with anti-β (1,3)-glucan antibody and PE-conjugated secondary antibody. The stained cells were measured by flow cytometry to quantify β (1,3)-glucan exposure. Strains were tested three times with 2 technical replicates each time, and statistical analysis was performed using one-way ANOVA. ****, *P* < 0.0001; *, *P* = 0.0142. Statistical comparisons between the *lrg1ΔΔste11ΔΔ*::*P_tetOFF_-STE11^ΔN467^* strain and the wild-type strain with or without doxycycline were done by ANOVA in the absence of the *lrg1ΔΔ* strain.

Our results indicate that Lrg1 negatively controls Cek1 phosphorylation through Cdc42 (Fig. 3), but it is not clear if the pathway acts through the canonical Cek1 MAPK cascade. Therefore, we next elucidated which upstream kinase cascade participates in this signal transduction pathway. The Ste11 MAPKKK sits at the top of the Cek1 MAPK module; therefore, we disrupted the *STE11* gene in the *lrg1ΔΔ* mutant to determine if this would prevent Cek1 activation. One *STE11* allele was deleted in the *lrg1ΔΔ* mutant by the use of SAT1-flipper (46). However, we could not disrupt the second *STE11* allele in the heterozygous mutant. Thus, we replaced the second allele with the hyperactive *STE11^ΔN467^* allele under the regulation of the tetracycline-repressible promoter (*P_tetOFF_*-*STE11^ΔN467^*). This resulted in a *lrg1ΔΔste11ΔΔ*::*P_tetOFF_*-*STE11^ΔN467^* strain, indicating that *STE11* is not essential in the *lrg1ΔΔ* background but that it may be difficult to recover under transformation conditions. The *lrg1ΔΔste11ΔΔ*::*P_tetOFF_-STE11^ΔN467^* mutant was treated with 0.5 μg/ml of doxycycline overnight to repress *STE11^ΔN467^* expression, followed by subculturing in fresh yeast extract-peptone-dextrose (YPD) medium with or without doxycycline for 3 h. Western blotting was performed to measure levels of Cek1 and Mkc1 activation. As shown in Fig. 4A, failure to express Ste11 in the *lrg1ΔΔ* mutant (*lrg1ΔΔste11ΔΔ*::*P_tetOFF_-STE11^ΔN467^* plus doxycycline) blocked Cek1 phosphorylation. This result indicates that Lrg1 negatively controls Cek1 activity through the Ste11 MAPKKK.

We next investigated if β (1,3)-glucan exposure would also be suppressed in the *lrg1ΔΔ* mutant when Ste11 expression was repressed, as we expected. Overnight cultures of *Candida* cells (with or without doxycycline) were stained with anti-β (1,3)-glucan antibody and then assessed by flow cytometry to quantify unmasking. As shown in Fig. 4B, inhibition of Ste11 expression by addition of doxycycline (*lrg1ΔΔste11ΔΔ*::*P_tetOFF_-STE11^ΔN467^* plus doxycycline) completely blocked β (1,3)-glucan unmasking. This indicates that hyperactivation of the Cek1 MAPK pathway was responsible for exposing β (1,3)-glucan in the *lrg1ΔΔ* mutant.

### Cells with a *lrg1ΔΔ* mutation or expressing the hyperactive *STE11^ΔN467^* allele induce TNF-α secretion from RAW264.7 macrophages.

**FIG 5:**
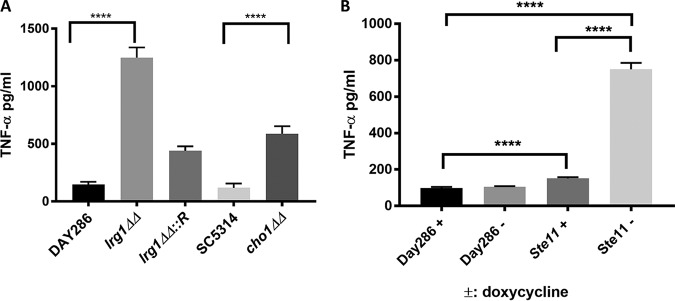
Conditions that hyperactivate the Cek1 pathway cause a significant increase in TNF-α secretion from macrophages compared to wild-type cells. (A) C. albicans cells were grown in YPD overnight at 30°C, subjected to UV inactivation, and then cocultured with RAW264.7 macrophages for 4 h. The RAW264.7 macrophage supernatant was collected and filtered through a 0.22-μm-pore-size syringe filter to remove cell debris. The filtrates were assayed by ELISA to quantify the TNF-α production. Samples were tested in triplicate three times (*n* = 9). The statistical analysis was done by one-way ANOVA. ****, *P* < 0.0001. (B) C. albicans wild-type and *P_tetOFF-_STE11^ΔN467^* strains were cultured overnight in YPD medium at 30°C (with or without doxycycline) and were then subjected to UV killing and incubated with RAW264.7 macrophages as described for panel A. ****, *P* < 0.0001. *lrg1ΔΔ*::*R *=* *reintegrated strain.

The relationship between greater β (1,3)-glucan exposure and increased TNF-α secretion has been studied intensively (9, 11, 13, 15, 17, 32). Due to the strong β (1,3)-glucan exposure exhibited by the *lrg1ΔΔ* mutant (Fig. 1), we performed enzyme-linked immunosorbent assays (ELISAs) to study if unmasking in the *lrg1ΔΔ* strain correlates with increased TNF-α production. As shown in Fig. 5A, loss of *LRG1* significantly upregulates TNF-α secretion from RAW264.7 murine macrophages. The *cho1ΔΔ* mutant represented in Fig. 5A served as a positive control, as it has been shown to cause upregulation of TNF-α production compared to the wild-type SC5314 strain (11, 15, 17).

### Overexpression of the hyperactive *STE11^ΔN467^* allele reduces virulence in mice.

Cek1 has been shown previously to undergo hyperactivation under multiple conditions associated with β (1,3)-glucan exposure, including caspofungin treatment (37), *CHO1* disruption (11), hyperactivation of Cdc42 and hyperactivation of Rho1 (17), and *LRG1* disruption (Fig. 1; see also Fig. 2). Furthermore, hyperactivation of Cek1 via an activated allele of *STE11* (*STE11^ΔN467^*) causes unmasking (17). We suspect that hyperactivation of the Cek1 MAPK pathway would lead to decreased virulence. Many of the mutants we have described such as the hyperactive Cdc42 or Rho1, *lrg1ΔΔ*, or *cho1ΔΔ* mutants are very pleiotropic, which prevents us from assessing the specific impact of hyperactivation of the Cek1 MAPK on virulence. However, the *STE11^ΔN467^* mutation causes a more specific upregulation of Cek1 activation (17). Thus, to test this *in vivo*, we overexpressed Ste11^ΔN467^ from the doxycycline-repressible promoter (*P_tetOFF_*) (47) so we could then use this in the mouse model of infection and turn the gene on or off in the host after infection. The *P_tetOFF_-STE11^ΔN467^* construct should show strong overexpression of the gene in the absence of doxycycline but should repress it in the presence of doxycycline (48). To test this, we transformed the *P_tetOFF_-STE11^ΔN467^* allele into wild-type *Candida* strain DAY286 and confirmed that Cek1 was hyperactivated in the absence of doxycycline (Fig. S3). Previous overexpression experiments performed with *STE11^ΔN467^* using the *P_MAL2_-STE11^ΔN467^* construct showed hyperactivation of Cek1 but not Mkc1. However, when we upregulated *STE11^ΔN467^* with the *P_tetOFF_-STE11^ΔN467^* construct, we observed strong hyperactivation of Cek1 and Mkc1. This may have occurred because a strong enough activation of Cek1 causes a compensatory activation of Mkc1 (17), although this is not certain.

10.1128/mBio.01767-19.4 FIG S3 Cek1 was hyperphosphorylated when the *P_tetOFF_-STE11^ΔN467^* construct was induced. The wild-type and *P_tetOFF_-STE11^ΔN467^* strains were cultured overnight in YPD medium at 30°C in the presence of doxycycline, washed with PBS, diluted to an OD_600_ of 0.2 in YPD medium with or without doxycycline, and grown for 3 h to the log phase. Western blotting was performed on the *Candida* cell extracts to evaluate the activation state of MAPKs. Anti-phospho-p44/42 antibody was used as the primary antibody to detect the levels of phosphorylated Cek1 and Mkc1 MAPKs, and anti-tubulin antibody was used as a control. Download FIG S3, TIF file, 0.3 MB. Copyright © 2019 Chen et al. 2019 Chen et al. This content is distributed under the terms of the Creative Commons Attribution 4.0 International license.

Immunofluorescent staining of the *P_tetOFF_*-*STE11^ΔN467^* strain for β (1,3)-glucan exposure indicates that under conditions of culture in YPD medium overnight without doxycycline, the mutant exhibited significantly increased β (1,3)-glucan exposure (Fig. S4). We also observed a modest increase in (1,3)-glucan exposure in the *P_tetOFF_*-*STE11^ΔN467^* strain with doxycycline, which may have been due to basal expression of the *P_tetOFF_*-*STE11^ΔN467^* allele even in the presence of the drug. To evaluate the correlation between β (1,3)-glucan exposure and TNF-α secretion in the *P_tetOFF_*-*STE11^ΔN467^* strain, we performed ELISAs on macrophages exposed to overnight cultures of wild-type DAY286 and the *P_tetOFF_*-*STE11^ΔN467^* strain in the presence or absence of doxycycline. As shown in Fig. 5B, when Ste11^ΔN467^ was induced in the *P_tetOFF_*-*STE11^ΔN467^* strain (without doxycycline), TNF-α production was significantly upregulated in RAW246.7 macrophages. TNF-α levels were also slightly elevated in the doxycycline-treated *P_tetOFF_-STE11^ΔN467^* strain, suggesting that the *STE11^ΔN467^* gene was basally expressed under this condition.

10.1128/mBio.01767-19.5 FIG S4 The *STE11^ΔN467^* strain exhibits increased β (1,3)-glucan exposure under conditions of induction. The *P_tetOFF_-STE11^ΔN467^* strain was cultured overnight in YPD medium at 30°C in the presence or absence of doxycycline. The overnight culture was stained with anti-β (1,3)-glucan antibody and phycoerythrin (PE)-conjugated secondary antibody. Flow cytometry was then performed to quantify the level of β (1,3)-glucan exposure. Samples were tested three times with two replicates each time. **, *P* = 0.0043; *, *P* = 0.0158. MFI, mean fluorescence intensity. These data are the same as those for the overnight (O/N) samples shown in Figure 8A, but are shown here alone for comparison. Download FIG S4, TIF file, 0.1 MB. Copyright © 2019 Chen et al. 2019 Chen et al. This content is distributed under the terms of the Creative Commons Attribution 4.0 International license.

### Hyperactivation of Cek1 results in decreased fungal virulence *in vivo*.

**FIG 6:**
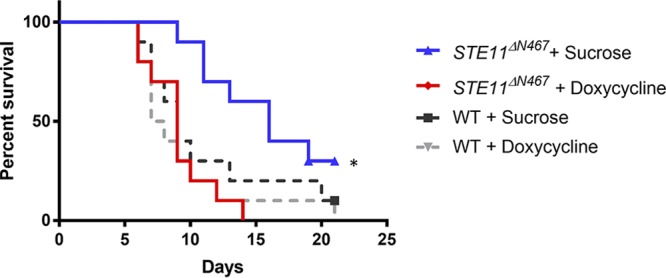
Hyperactivation of Cek1 leads to attenuated fungal virulence in a mouse model of systemic infection. C. albicans strains that were wild type (DAY286) or carried the *P_tetOFF_-STE11^ΔN467^* allele were cultured overnight at 30°C with doxycycline to repress Ste11^ΔN467^ expression. The overnight cultures were diluted to 10^6^ cells/ml, and 100-μl volumes of the suspensions were injected into the lateral tail veins of outbred ICR mice. Mice were given 5% sucrose in their drinking water either without doxycycline or with 2 mg/ml of doxycycline to repress Ste11^ΔN467^ expression *in vivo*. The symptoms of illness were monitored over 21 days. Each group had 10 mice. *, *P* = 0.016.

We predicted that strong expression of the *STE11^ΔN467^* allele in mice would lead to a loss of virulence, due to increased unmasking. To test this, the wild-type and *P_tetOFF_-STE11^ΔN467^* strains were cultured in YPD overnight with doxycycline to repress Ste11^ΔN467^ expression and were then injected into the tail vein of outbred ICR mice. The mice that were injected with the *P_tetOFF_*-*STE11^ΔN467^* strain and provided with doxycycline succumbed to fungal infection in ∼10 days. This is similar to the results seen with mice injected with the wild-type strain with or without doxycycline. However, the mice that were infected with the *P_tetOFF_*-*STE11^ΔN467^* strain and that did not receive doxycycline (where *STE11^ΔN467^* is strongly expressed and induces activation of Cek1) (Fig. S3) survived significantly longer than mice in the other groups (Fig. 6).

**FIG 7:**
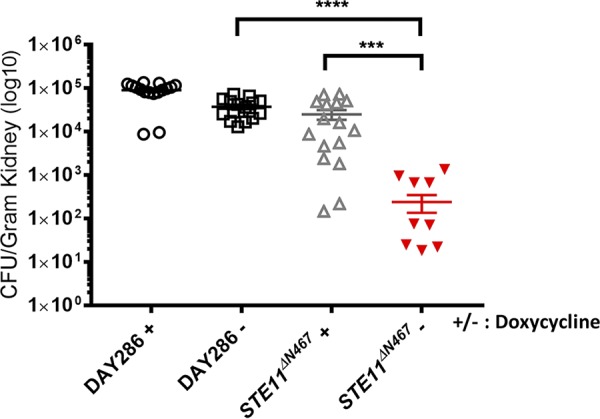
Ste11 hyperactivation causes decreased kidney fungal burden in the mouse model of systemic infection. C. albicans DAY286 (wild-type) and *P_tetOFF_*-*STE11^ΔN467^* strains were cultured overnight at 30°C with doxycycline to repress Ste11^ΔN467^ expression. The overnight culture was diluted to 10^6^ cells/ml, and 100 μl of the suspension was injected to the lateral tail vein of outbred ICR mice. Mice were given drinking water mixed with 5% sucrose with 2 mg/ml of doxycycline to repress Ste11^ΔN467^ expression *in vivo* or without doxycycline to promote Ste11^ΔN467^ expression. The mice were sacrificed 4 days postinfection, and the kidneys were removed from each mouse and homogenized. The homogenates were diluted to 10^−3^ by performing serial dilutions. The diluted homogenates (1 ml) from each dilution were plated on the YPD plates and cultured at 30°C for 2 days. The CFU were counted on each plate. Eight mice were tested for each strain. ***, *P* = 0.0006; ****, *P* < 0.0001.

Furthermore, when mice were sacrificed at 4 days postinfection, mice infected with the *P_tetOFF_*-*STE11^ΔN467^* strain that did not receive doxycycline exhibited (2.43 ± 1.07) ×10^2^ CFU g^−1^ kidney, while the mice infected with the same strain that did receive doxycycline exhibited (2.51 ± 0.65) ×10^4^ CFU g^−1^ kidney (Fig. 7). Thus, there was a decrease of ∼2 log (*P* = 0.0006) in kidney fungal burden when the gene was expressed. The growth of a strain expressing the *STE11^ΔN467^* allele from the maltose promoter is not affected under those conditions (17), but we wanted to determine if growth of the mutant might be strongly attenuated by activating expression of *STE11^ΔN467^* from the *P_tetOFF_*-promoter. Therefore, a growth curve was measured *in vitro* (Fig. S5A). This demonstrated that the *P_tetOFF_-STE11^ΔN467^* strain showed an increase in its doubling time between 6 and 8 h of the growth curve in the absence of doxycycline versus in the presence of the drug (90 versus ∼103 min). Between 8 and 10 h, the doubling times seem to have been similar, at around 95 to 100 min for both strains, and the strains grew to similar densities by 24 h. The *P_tetOFF_-STE11^ΔN467^* strain grew slower than the wild-type strain, and the differences were stronger under conditions lacking doxycycline. Altogether, it appears from the growth curves that growth was slower following induction of the gene but that the strain might have begun to recover over time. The impact of growth on virulence is difficult to assess in comparisons of results determined under *in vitro* conditions to those seen under *in vivo* conditions since mutants that show stronger growth defects *in vitro*, such as the *psd1ΔΔpsd2ΔΔ* mutant, have been shown to have no defects in virulence (49).

10.1128/mBio.01767-19.6 FIG S5 The growth of the *P_tetOFF_*-*STE11^ΔN467^* strain was monitored under different conditions. (A) Cells were grown overnight in YPD medium at 30°C plus doxycycline, diluted to an OD_600_ of 0.1, and transferred to fresh YPD medium with or without doxycycline. A growth curve was generated with three replicates for each condition. Optical densities of the *P_tetOFF_-STE11^ΔN467^* strain were compared under conditions that included or did not include doxycycline at each time point using two-way ANOVA (**, *P* = 0.0024; ****, *P* < 0.0001). (B) Cek1 hyperactivation does not result in susceptibility to cell wall inhibitors. Ten-fold dilutions of overnight cultures of the wild-type strain and the *STE11^ΔN467^* strain were spotted onto YPD media with different cell wall inhibitors as indicated and grown for 2 days. The medium was maintained with or without 0.5μg/ml of doxycycline (used to control Cek1 activation). Download FIG S5, TIF file, 1.4 MB. Copyright © 2019 Chen et al. 2019 Chen et al. This content is distributed under the terms of the Creative Commons Attribution 4.0 International license.

Another concern is that the expression of the Ste11^ΔN467^ allele might cause overall cell wall damage. To test this, we examined if expression of the *STE11^ΔN467^* gene (without doxycycline) correlated with hypersensitivity to cell wall stress. We tested strains for growth in the presence of the cell wall stressors calcofluor white, SDS, and Congo red. However, the *P_tetOFF_*-*STE11^ΔN467^* strain did not show differential sensitivity to any of these compounds (Fig. S5B). Thus, we suspect that activation of the *P_tetOFF_*-*STE11^ΔN467^* allele decreases virulence by activating the host immune response, although this remains to be tested more carefully.

### β (1,3)-Glucan exposure induced by hyperactivation of the Cek1 cascade correlates with upregulation of cell wall synthesis genes.

**FIG 8:**
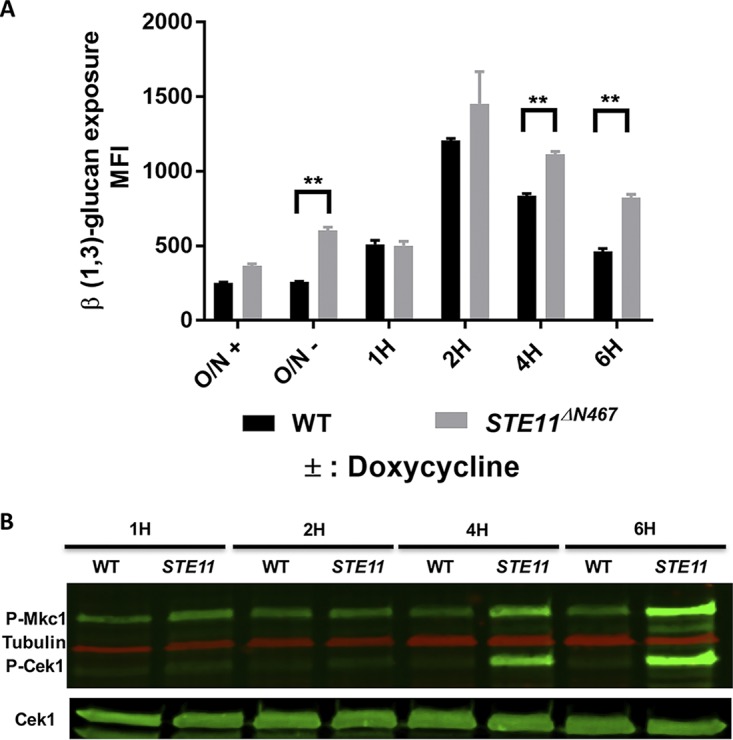
The *P_tetOFF_-STE11^ΔN467^* strain exhibited β (1,3)-glucan exposure in a time-dependent manner in the absence of doxycycline. (A) The wild-type and *P_tetOFF_-STE11^ΔN467^* strains were cultured overnight in YPD medium at 30°C in the presence or absence of doxycycline. Aliquots of the overnight cultures (O/N+ and O/N–) were taken as 0 time point controls and stained with mouse anti-β (1,3)-glucan antibody and goat-anti-mouse PE-conjugated secondary antibody. Aliquots of the overnight cultures of both wild-type and *P_tetOFF_*-*STE11^ΔN467^* strains that had been grown with doxycycline were washed three times with PBS and diluted to an OD_600_ of 0.2 in fresh YPD without doxycycline (to switch on Ste11^ΔN467^ expression) and then grown for 1 h, 2 h, 4 h, and 6 h. Cells were collected at the indicated time points and stained as described above. All the stained cells were analyzed using the flow cytometer to quantify the β (1,3)-glucan exposure level. Samples were tested three times with 2 replicates each time. **, *P* < 0.008. (B) Cells of wild type (WT) and the *P_tetOFF_-STE11^ΔN467^* strain (*STE11*) were grown overnight with doxycycline, diluted as described for panel A without doxycycline, and grown to specific time points, and then total protein was isolated. Western blotting was performed on the isolated protein to evaluate MAPK phosphorylation by using anti-phospho-p44/42 antibody and Cek1 and tubulin antibodies as controls.

A full model to describe how β (1,3)-glucan exposure is mediated at the molecular level within the cell wall has not yet been developed, although a growing body of evidence indicates that it correlates with activation of cell wall repair processes (10, 16, 17). To investigate how Cek1 activation could contribute to this, the gene expression profile resulting from hyperactivated Cek1 was measured. The *P_tetOFF_*-*STE11^ΔN467^* strain with or without doxycycline was compared to the isogenic wild-type strain under identical conditions. To determine the time point when the *P_tetOFF_*-*STE11^ΔN467^* strain would start to display β (1,3)-glucan exposure, we cultured the *Candida* cells overnight with doxycycline to repress Ste11^ΔN467^ expression, followed by subculturing in fresh YPD medium without doxycycline to switch on the Ste11^ΔN467^ expression for up to 6 h. The *Candida* cells were then subjected to immunofluorescence staining using the anti-β (1,3)-glucan antibody, and flow cytometry to quantify the exposure level over time. As shown in Fig. 8A, the *P_tetOFF_*-*STE11^ΔN467^* strain began to display significant unmasking compared to the wild-type strain at 4 h after doxycycline was removed from the medium. Both strains exhibited unmasking at 2 h, but the level diminished over the next 2 h more quickly in the wild-type strain than in the *P_tetOFF_*-*STE11^ΔN467^* strain. Interestingly, the earliest time point at which unmasking was significantly different between the *P_tetOFF_*-*STE11^ΔN467^* strain and wild type was 4 h, which correlates well with Western blotting results revealing that Cek1 began to display clearly increased phosphorylation 4 h after doxycycline was removed (Fig. 8B).

Based on these timing parameters, we grew cells overnight with doxycycline and then extracted total RNA from the wild-type and *P_tetOFF_*-*STE11^ΔN467^* strains 4 h after subculture under conditions with or without doxycycline, with each condition represented by three biological replicates (12 total samples). RNA sequencing was performed using an Illumina MiSeq platform, which generated a total of 59 million 75-bp paired-end reads from 12 libraries. A principal-component-analysis (PCA) plot was created by using the RNA Analysis Package in CLC Genomics Workbench software to determine the variability between replicates and different treatment samples. As shown in Fig. S6A, the two wild-type groups (with or without doxycycline) clustered tightly, suggesting that doxycycline does not cause detectable effects on the wild-type strain. The *P_tetOFF_*-*STE11^ΔN467^* strain was found in two distinct clusters based on the presence and absence of doxycycline, but both were separated from the wild type. The *P_tetOFF_*-*STE11^ΔN467^* strain assayed in the presence of doxycycline grouped away from the wild-type strain, and this might have been because of basal expression of Ste11^ΔN467^, which would explain the modest increase in unmasking and levels of TNF-α elicited from macrophages by these cells compared to the wild-type results, even in the presence of doxycycline (Fig. 5B; see also Fig. S4). The *P_tetOFF_*-*STE11^ΔN467^* strain assayed in the absence of doxycycline grouped even further away from the wild-type strain but was also separated from the *P_tetOFF_*-*STE11^ΔN467^* strain assayed in the presence of doxycycline, indicating that the presence or absence of doxycycline strongly influenced gene expression in this strain. To be certain that the expression levels of genes were consistent overall between the strains and that the PCA differences were not due to anomalies associated with overall sample preparation, we examined 5 housekeeping genes of known expression levels and found that they were highly consistent between the samples (Fig. S6B).

10.1128/mBio.01767-19.7 FIG S6 PCA plot and housekeeping gene expression among samples. (A) A PCA plot was created by using the RNA Analysis Package in the CLC Genomics Workbench software package (V12.0) to determine levels of variability between replicates and different treatment samples. Original symbols from CLC were highlighted using Powerpoint to increase visibility. (B) The TPM (total per million) reads of five housekeeping genes (*TEF1*, *ACT1*, *TUB1*, *ENO1*, and *PMA1*) were used to calculate the average for each condition to determine if overall gene expression values were consistent between samples. Download FIG S6, TIF file, 0.3 MB. Copyright © 2019 Chen et al. 2019 Chen et al. This content is distributed under the terms of the Creative Commons Attribution 4.0 International license.

**FIG 9:**
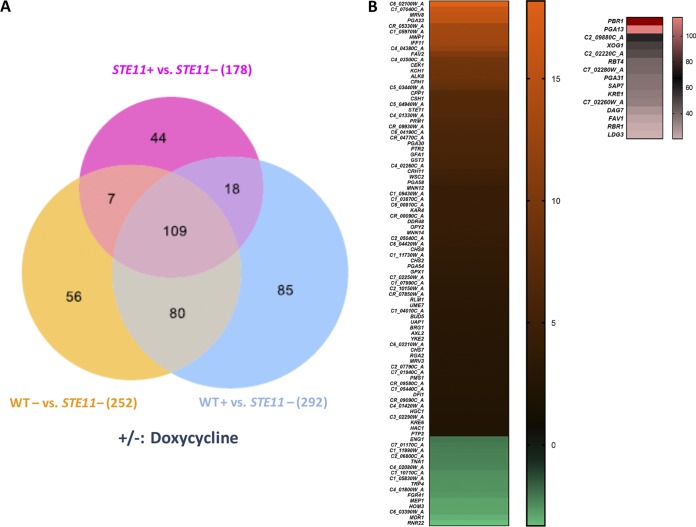
Genes that were differentially expressed under *STE11^ΔN467^*-inducing condition were enriched for cell wall repair genes. (A) Venn diagram showing that 109 genes were differentially expressed in the *P_tetOFF_*-*STE11^ΔN467^* strain when Cek1 is induced (*STE11* without [−] doxycycline) compared to the other three control groups (the wild-type strain with or without doxycycline and STE11 with doxycycline). Genes with a false-discovery rate (FDR) adjusted *P* value of *<*0.05 were considered differentially expressed. (B) Heat maps were generated for genes with expression values exceeding a factor of 2 (fold change) under *STE11^ΔN467^* induction conditions (*STE11*–doxycycline) compared to the other three controls.

Under the *STE11^ΔN467^*-inducing conditions (in the absence of doxycycline), there were 109 genes that were differentially regulated as shown by a >2-fold change in expression value compared to the other three conditions (wild-type strain with or without doxycycline and *STE11^ΔN467^* strain with doxycycline) (Fig. 9A). Among the 109 genes, 16 genes were downregulated and 93 genes were upregulated, and these genes are listed in Table S1 in the supplemental material. Although Cek1 activation is associated with hyphal formation under some conditions (50), there are only a few genes that encode conventional hyphal-specific proteins. A number of genes commonly associated with hyphal formation, such as members of the Als family and Ece1, were not upregulated. However, 13 genes that are involved in cell wall construction were upregulated (Fig. 9B; see also Table 1). The 93 upregulated genes are enriched for cell wall or extracellular genes. For example, 14% of the upregulated genes in our data set encode secreted proteins, whereas only 5% of the genes in the total *Candida* genome encode secreted proteins. This indicates that there is an ∼2-fold enrichment in cell wall genes in our data set. A total of 26 genes, including *OPY2* and *RBT4*, are responsive to chemicals and stress, and 10 genes encode signal transduction proteins (*CPP1*, *BUD5*, *RGA2*, *HAC1*, *WSC2*, *CPH1*, etc.). In the category of downregulated genes, it is noteworthy that Eng1 expression was decreased ∼2-fold under conditions of *STE11^ΔN467^* induction. Eng1 is an endo-β (1,3)-glucanase and has been reported to keep glucan masked by removing the exposed β (1,3)-glucan in the fungal pathogen Histoplasma capsulatum. Taken together, these data suggest that β (1,3)-glucan unmasking could be caused by Cek1 hyperactivation through inappropriate expression of cell wall repair machinery.

**TABLE 1:** Cell wall and stress genes induced by *STE11ΔN467* mutant without doxycycline versus the wild-type strain with or without doxycycline and *STE11^ΔN467^* with doxycycline

Gene ontology term	Names
Function	
Cell wall organization	CEK1, CHS2, CPH1, CRH11, ENG1,^*a*^ HAC1, HWP1, KRE6, PGA13, PGA31, RLM1, WSC2, XOG1
Stress/chemical response	BRG1, C1_03870C_A, C1_10710C_A,^*a*^ C3_02290W_A, CEK1, CPH1, CPP1, DDR48, FGR41,^*a*^ GPX1, HAC1, MDR1,^*a*^ MEP1, OPY2, PMS1, RLM1, STE11, WSC2, C7_01170C_A,^*a*^ DAG7, GFA1, PGA23, PGA31, RBT4, RLM1, TNA1^*a*^

Component	
Cell wall and extracellular regions	CRH11, CSH1, DDR48, ENG1,^*a*^ HWP1, IFF11, KRE1, PGA13, PGA30, PGA31, XOG1, C4_01800W_A,^*a*^ DAG7, FGR41,^*a*^ RBT4, SAP7

a Transcriptionally downregulated gene.

10.1128/mBio.01767-19.9 TABLE S1 Genes differentially expressed when *STE11^ΔN467^* is overexpressed. Download Table S1, DOCX file, 0.1 MB. Copyright © 2019 Chen et al. 2019 Chen et al. This content is distributed under the terms of the Creative Commons Attribution 4.0 International license.

In addition, our data suggest that Cph1 is likely responsible for these effects. Cph1 is known to act downstream of Cek1 and is upregulated 8.4-fold under *STE11^ΔN467^* induction conditions compared to that of the wild type (Fig. 9B). Cph1 is important for C. albicans filament development on certain solid media (51) and is involved in switching from white cells to mating-competent opaque cells (52). Thus, Cph1 may be a target through which Cek1 acts to cause unmasking. Alternatively, the transcription factor Ace2, which is involved in cell wall glycosylation, morphogenesis, and virulence (53, 54), could be a target through which Cek1 acts to cause unmasking. Ace2 does not display altered transcripts when Cek1 is hyperactivated, but this does not mean that it is not playing a role in unmasking. In fact, both transcription factors could potentially play a role in this phenotype.

## DISCUSSION

Previously, our laboratory determined that hyperactivation of Cek1 promotes β (1,3)-glucan exposure in response to loss of phosphatidylserine synthase (17). This process involves the activation of the GTPase Cdc42, which positively regulates Cek1 MAPK activity (17). In this communication, we characterized a novel upstream regulator of Cek1 by revealing that the Lrg1 GAP negatively regulates Cek1 activity (Fig. 2; see also Fig. 3). Our data suggest that Lrg1 acts through the GTPase Cdc42, but not the GTPase Rho1, which has been reported as a target of Lrg1 in the literature. Lrg1 disruption stimulates significantly increased β (1,3)-glucan unmasking, which further induces a higher level of TNF-α secretion from murine macrophages (Fig. 1; see also Fig. 5).

### The Lrg1-Cdc42 cascade modulates Cek1 MAPK activity via Ste11 in C. albicans.

In S. cerevisiae, Lrg1 has been shown to interact with the activated form of Rho1 (GTP-Rho1) and act as a GAP (25, 55). However, there are discrepancies concerning the negative regulation of other GTPases by Lrg1 in different studies. Roumanie et al. found that Lrg1 acts as GAP for two other GTPases, Rho2 and Cdc42 (56). In contrast, Fitch et al. found that Lrg1 is a Rho1-specific GAP *in vitro* by measuring the amount of Rho1 bound to [α-^32^P]-labeled GDP or GTP, after stimulation with purified Lrg1 (25). Regarding the effect of Lrg1 on Rho1-mediated cell wall processes, Lrg1 is reported to negatively regulate glucan synthase activity, while there is controversy with regard to its role in activation of the cell wall integrity Mkc1 pathway (38, 55). These opposing results could have been a consequence of the different backgrounds of S. cerevisiae strains used. Lrg1 was shown previously to serve as a Rho1-specific GAP and impact several downstream pathways of Rho1 in Neurospora crassa (39). In C. albicans, the *LRG1* disruption was reported to increase hyphal formation and biofilm development, which are phenotypes shared with a *RHO1^Q67L^* gain-of-function mutant. In addition, the *lrg1*∆∆ hyperfilamentation phenotype is blocked by a mutation in the Rho1 effector PKC1; however, there was no biochemical evidence indicating that Lrg1 acts as Rho1 GAP (24, 57).

In this communication, we provide evidence that loss of Lrg1 in C. albicans increases β (1,3)-glucan unmasking (Fig. 1) and that this is likely mediated by hyperactivated Cek1 (Fig. 2). Our results also suggest that Lrg1 does not act as a Rho1 GAP in C. albicans. Lrg1 does not show Rho1 inhibitory activity *in vivo*, nor does it inhibit Rac1 activity (Fig. 3; see also Fig. S1). Instead, Lrg1 exhibits repression of the activities of both Cdc42 and Ras1, as indicated by significant induction of both GTPase activities *in vivo* in the *lrg1ΔΔ* mutant (Fig. 3). Previous work showed that Pkc1 is epistatic to *Lrg1* (24). Thus, Pkc1 signaling is necessary for *lrg1*∆∆-driven filamentation, but it is possible that Pkc1 acts in parallel to the Lrg1 activated pathway(s) rather than directly downstream. The unexpected results imply that signal rewiring occurs in the pathogenic C. albicans species versus the nonpathogenic S. cerevisiae species. Ras1 plays pivotal roles in fungal morphogenesis and, as a result, contributes to virulence (35). Loss of *RAS1* causes defects in the yeast-to-hyphal transition, which nonetheless can be reversed by overexpression of signaling components in the Cek1 pathway in the *ras1ΔΔ* mutant (35). Although this suggests that Ras1 might be located upstream of Cek1, we cannot rule out the possibility that Cek1 and Ras1 act in parallel manners. Our Western blotting results show that a hyperactive *RAS1^G13V^* mutant does not display hyperactivated Cek1 (Fig. 3E; see also Fig. S2), suggesting that hyperactivation of Ras1 is not sufficient to induce Cek1 phosphorylation and that these two proteins are not in a linear signaling cascade in these conditions.

**FIG 10:**
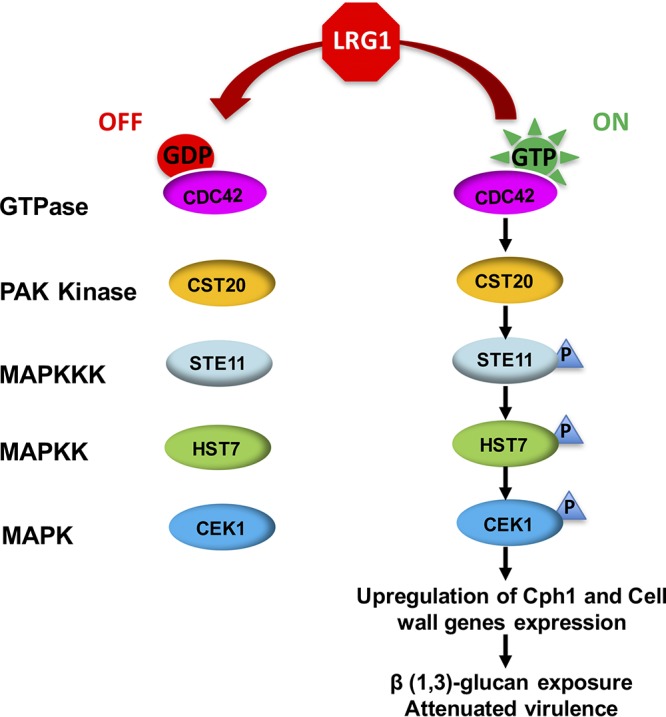
A model for how Lrg1 acts as a molecular switch to regulate Cek1 activity negatively via the GTPase Cdc42 and the Ste11 MAPKKK. In this model, Lrg1 acts as a GAP for the GTPase Cdc42 and therefore increases the GDP-bound state to suppress Cek1 phosphorylation (activation). Therefore, Lrg1 disruption increases Cdc42 activity *in vivo* by increasing the level of GTP-bound Cdc42. This activates the canonical downstream Ste11-Hst7-Cek1 cascade. Hyperactivated Cek1 was shown in this study to stimulate β (1,3)-glucan exposure potentially via transcriptional upregulation of transcription factor Cph1 and expression of cell wall genes, and this then compromises fungal virulence.

The Ste11 MAPKKK is a well-known upstream regulator of Cek1 activity, and the hyperactivated *STE11^ΔN467^* mutant causes β (1,3)-glucan exposure in C. albicans (17). Here we show that Ste11 is also involved in the Lrg1-dependent Cek1 hyperactivation, given that repression of *STE11* expression rescued both β (1,3)-glucan exposure and Cek1 hyperphosphorylation in the *lrg1ΔΔ* mutant (Fig. 4). Given the established effect of Cdc42 on Cek1 activation (17), our results indicate that Lrg1 negatively modulates Cek1 activity via the GTPase Cdc42 which acts through the Ste11 MAPKKK (Fig. 10).

However, the results presented in Fig. 4 did yield a result that was somewhat difficult to explain. The results of comparisons of the *lrg1ΔΔste11ΔΔ*::*P_tetOFF_-STE11^ΔN467^* strain with doxycycline to the *lrg1ΔΔ* strain and wild-type strain are clear and reveal that the *lrg1ΔΔ* strain acts through Ste11. The surprising result was that the *lrg1ΔΔste11ΔΔ*::*P_tetOFF_-STE11^ΔN467^* strain without doxycycline exhibited a much lower level of Cek1 activation and β (1,3)-glucan exposure than the *lrg1ΔΔ* mutant (Fig. 4). The reason for this was not clear, but it was hypothesized that the level of *STE11* gene expression might normally be increased during activation of Cek1 MAPK pathway, since we saw *CEK1*, *STE11*, and *CPH1* gene expression increased when Cek1 was activated (in the absence of doxycycline) in a strain that was of the genotype *STE11/ste11Δ*::*P_tetOFF_-STE11^ΔN467^* (Fig. 9B). These data indicate that the activated Cek1 pathway normally activates some of its own components transcriptionally in a positive-feedback loop. But, given these data, it would also suggest that this loop requires the presence of at least one wild-type allele of *STE11.* This is suggested because both the *lrg1ΔΔ* and *STE11/ste11Δ*::*P_tetOFF_-STE11^ΔN467^* strains have at least one copy of wild-type *STE11*, whereas the *lrg1ΔΔ ste11ΔΔ*::*P_tetOFF_-STE11^ΔN467^* strain has only a truncated copy of *STE11*. If our suppositions are correct and *STE11* is autoamplified in the presence of a wild-type copy, then we would expect to see a large increase in *STE11* expression in the *lrg1ΔΔ* mutant compared to the *lrg1ΔΔste11ΔΔ*::*P_tetOFF_-STE11^ΔN467^* strain with or without doxycycline. Surprisingly, quantitative reverse transcriptase PCR (qRT-PCR) revealed that the levels of *STE11* expression were not significantly different between the *lrg1ΔΔ* and the wild-type strain and even the *lrg1ΔΔ ste11ΔΔ*::*P_tetOFF_-STE11^ΔN467^* strain with doxycycline (see Fig. S7 in the supplemental material). In contrast, in the *lrg1ΔΔste11ΔΔ*::*P_tetOFF_-STE11^ΔN467^* strain without doxycycline there was an ∼3-fold overexpression of *STE11* (Fig. S7). The *P_tetOFF_-STE11^ΔN467^*-plus-doxycycline strain still expressed *STE11*, and this was likely because it was leaky in the presence of doxycycline (Fig. 5B; see also Fig. S4 and S6). Thus, the difference in behavior between the *lrg1ΔΔste11ΔΔ*::*P_tetOFF_-STE11^ΔN467^* and *lrg1ΔΔ* strains cannot be explained on the basis of *STE11* gene expression. Instead, it may be explained by differences in signaling at the protein level. The *lrg1ΔΔste11ΔΔ*::*P_tetOFF_-STE11^ΔN467^* strain exclusively expresses a form of *STE11* that is truncated for the inhibitor region which also includes Ste11 regulation sites (58). Thus, there is likely a positive-feedback loop for signaling that can be driven by *STE11^ΔN467^* only if there is a wild-type copy present to mediate the loop. This model is currently being explored.

10.1128/mBio.01767-19.8 FIG S7 *STE11* transcription level in different *Candida* strains. RNA was extracted from multiple *Candida* strains followed by reverse transcription to make cDNA. *STE11* expression was determined by performing quantitative PCR. The actin transcription level was quantified as a reference for each strain. The *STE11* transcription level is indicated as a ratio to the result determined with the wild type (WT) control after being normalized to actin transcripts for each strain. *, *P* value of <0.025 compared to all other strains. Download FIG S7, TIF file, 0.1 MB. Copyright © 2019 Chen et al. 2019 Chen et al. This content is distributed under the terms of the Creative Commons Attribution 4.0 International license.

### Central role of Cek1 activity in controlling β (1,3)-glucan masking.

The *STE11^ΔN467^* allele causes β (1,3)-glucan exposure when induced. Our RNA sequencing (RNA-Seq) results reveal that several categories of genes were expressed differentially when Ste11 and Cek1 were hyperactivated, and they included cell wall genes, signal transduction genes, and stress/chemical response genes (Fig. 9). Cph1, one of two well-known downstream transcription factors phosphorylated/activated by Cek1, was significantly upregulated under *STE11^ΔN467^* induction conditions (Fig. 9B). Cph1 is important for C. albicans filament development (51) and for white-to-opaque switching (52). A mutant strain that overexpresses Cph1 under yeast-inducing conditions induces pseudohyphal growth and expression of several hyphal-specific genes (59). Lowman et al. reported that C. albicans hyphal glucan exhibits a novel glucan structure in which hyphal glucan has a unique cyclical structure which intrinsically displays higher levels of β (1,3)-glucan exposure and immunogenicity (60). Our data show that *CPH1* was induced ∼9-fold, and it is possible that the β (1,3)-glucan structure might transition to a more extensively hyphal glucan structure even under yeast conditions and might therefore display more glucan exposure. Alternatively, changes in exposure may be related to aberrantly induced cell wall repair machinery. Cek1 is upregulated in response to caspofungin treatment (37, 61), which damages the cell wall; thus, Cek1 activation may contribute to unmasking caused by this drug as it upregulates cell wall repair genes.

Although Cph1 may be involved, it is also possible that Ace2 plays a role in Cek1-driven unmasking of β (1,3)-glucan. Ace2 is another downstream transcription factor of Cek1, and it is required for proper cell wall glycosylation and causes β (1,3)-glucan exposure when disrupted (53, 62), which is a phenotype shared with a *cek1ΔΔ* mutant (32). Ace2 is not induced transcriptionally when Cek1 is hyperactivated, but it may still be activated by Ste11 to contribute to unmasking. It is also possible that both Ace2 and Cph1 play a role in inducing β (1,3)-glucan exposure.

### β (1,3)-Glucan exposure correlates with decreased fungal virulence when Cek1 is hyperactivated.

Several cell wall-defective mutants, including *cho1ΔΔ*, *cek1ΔΔ*, *kre5ΔΔ*, and *phr1ΔΔ* mutants, have been found to exhibit attenuated fungal virulence in the mouse model of systemic infection (49, 50, 54, 63, 64). However, all of these mutants display pleotropic phenotypes. Therefore, it is hard to differentiate the effect of β (1,3)-glucan exposure on virulence in light of their other defects. Also, β (1,3)-glucan in these mutants is exposed even before the fungal cells are injected into the mouse. This raises the problem that the mutants with increased β (1,3)-glucan exposure might be recognized and cleared by the immune systems more rapidly, even before the systemic infection is established.

In this study, we made a *P_tetOFF_*-*STE11^ΔN467^* strain that addressed some of the disadvantages mentioned above. Ste11 sits upstream of Cek1 in C. albicans (50), and overexpression of *STE11^ΔN467^* causes greater induction of Cek1 activity and a greater level of β (1,3)-glucan exposure (17) (Fig. S3; see also Fig. S4). The *P_tetOFF_* promoter can be further used to control Ste11^ΔN467^ expression by adding/removing the inhibitor doxycycline as needed so that it can be used in animal studies (47, 48, 65). The *STE11^ΔN467^* mutant, when induced to hyperactivate Cek1, has decreased virulence *in vivo*, reflected by significantly greater mouse survival over 21 days compared to other control groups, and an ∼100-fold decrease in fungal burden in mouse kidneys (Fig. 6; see also Fig. 7). This suggests that the exposed β (1,3)-glucan might contribute to the attenuated fungal virulence, due to its higher visibility to immune receptors such as Dectin-1. There are alternative possible explanations as well, such as more-general defects in the cell wall, although this strain did not reveal hypersensitivity to general cell wall stressors such as SDS, calcofluor white, or Congo red (Fig. S5B). Regardless of the mechanism, virulence is compromised, and thus, hyperactivation of Cek1 may be useful for improving immune recognition of C. albicans and for adjunctive therapy.

## MATERIALS AND METHODS

### Strains and growth media.

The medium used to culture strains was yeast extract-peptone-dextrose (YPD) medium (1% yeast extract, 2% peptone, and 2% dextrose) (Thermo Fisher Scientific).(66) YPM medium (1% yeast extract, 2% peptone, 2% maltose [Thermo Fisher Scientific]) was used to flip out the SAT1-flipper cassette (46), which is under the control of maltose (*MAL2*) promoter. Doxycycline (Sigma-Aldrich, USA) was added at the working concentration of 0.5 μg/ml as the repressor for the tetracycline-repressing promoter.

### Strain construction.

Cloning procedures were performed following standard protocols. Plasmid and strain construction are described in Text S1 in the supplemental material.

10.1128/mBio.01767-19.1 TEXT S1 Strain construction. Download Text S1, DOCX file, 0.1 MB. Copyright © 2019 Chen et al. 2019 Chen et al. This content is distributed under the terms of the Creative Commons Attribution 4.0 International license.

### Protein purification.

To measure Rho1 activity, we generated a GST-RID-6×His (Rho1 interactive domain) construct that binds with GTP-Rho1 and expressed it in Escherichia coli strain BL21. The overnight culture was diluted by 1:100 in fresh LB medium and grown to an optical density at 600 nm (OD_600_) of 0.6 to 0.9. Then, IPTG (isopropyl-β-d-thiogalactopyranoside; Sigma-Aldrich) was added to reach a final concentration of 5 mM to induce GST-RID expression at 20°C for 20 h. The culture was pelleted by centrifugation and resuspended in cell lysis buffer (50 mM Tris-HCl, 500 mM NaCl, 30 mM imidazole, 400 μl of 0.25 mM phenylmethylsulfonyl fluoride [PMSF], 100 μl of β-mercaptoethanol [BME], 10 mM MgCl_2_, and 1 protease inhibitor tablet [Roche Diagnostics GmbH, Mannheim, Germany]). The suspension was agitated by sonication (Sonic Dismembrator F550 ultrasonic homogenizer; Fisher Scientific). The liquid was centrifuged for 1 h at 17,000 rpm at 4°C. The protein is soluble and was thus located in the supernatant. The solution was then slowly run through a nickel-nitrilotriacetic acid (Ni-NTA) column (Qiagen Inc., Germany) for binding, and the beads were then gently washed with wash buffer (50 mM Tris-HCl [pH 8.0], 500 mM NaCl, 30 mM imidazole) to remove the nonspecific binding protein. To elute out the His-tagged protein, 6 rounds of 250 μl of elution buffer (the same as the wash buffer except with 300 mM imidazole) were added. The eluted fractions were then run through a PD-10 column (GE Healthcare) to remove the imidazole. The product was applied to an Amicon Ultra 0.5-ml centrifugal filter unit (Merck KGaA, Darmstadt, Germany) to concentrate the protein.

### Western blotting.

Western blotting was performed as previously described (17). To detect the phosphorylation of Cek1 and Mkc1 MAPKs, rabbit anti-phospho-p44/42 antibody (Cell Signaling Technology, Inc., USA) was utilized at a 1:2,000 dilution. The expression of total Mkc1 was detected with the primary rabbit anti-Mkc1 Ab at a 1:1,000 dilution. The level of expression of total Cek1 was measured with a rabbit anti-Cek1 Ab at a 1:1,000 dilution. The secondary antibody against phospho-p44/42 Ab, Mkc1 Ab, and Cek1 Ab was IRye800CW goat anti-rabbit IgG (H+L) conjugate (Li-Cor Biosciences) (green; 1:10,000 dilution). Tubulin was detected as a control with rat anti-tubulin primary antibody (Bio-Rad Laboratories Inc., USA) (1:1,000 dilution) and IRDye 680RD goat anti-rat IgG (H+L) (Li-Cor Biosciences) (red; 1:10,000 dilution).

### Pulldown assay for active GTPases.

The GTPase activity assay was performed as previously described (17). To detect Cdc42 activity, 1,500 μg of total protein was used for the pulldown procedure following the instructions from an Active Cdc42 pulldown and detection kit (Thermo Fisher Scientific, USA). The same kit was used to pull down the active GTP-bound GFP-tagged-Rac1 (see “Strain construction” above), since the GST-Pak1 provided in the kit can also bind active Rac1. GTPase Ras1 activity was evaluated following the protocol from an Active Ras1 pulldown and detection kit (Thermo Fisher Scientific, USA). To pull down the active GTP-bound c-*myc*-tagged Rho1 (see “Strain construction” above), an Active Rho pulldown and detection kit (Thermo Fisher Scientific, USA) was purchased and the instructions were followed, except that the purified GST-Rhotekin-RBD peptides provided in the kit were replaced with the purified GST-RID-6×His peptides that we generated (see “Protein purification” above) for optimization of binding between GTP-Rho1 and the RID domain.

The antibody used to detect Cdc42 was rabbit polyclonal anti-S. cerevisiae Cdc42 (sent by Doug Kellogg at the University of California, Santa Cruz.). The antibody to detect Ras1 was mouse monoclonal anti-Ras1 antibody (anti-Ras, clone RAS10; Millipore Sigma, USA) with the working concentration at 1.5 μg/ml. The antibody for c-*myc*-Rho1 detection was mouse anti-*c*-Myc monoclonal antibody (9E10) (Thermo Fisher Scientific, USA) at a 1:1,000 dilution. The rabbit anti-GFP antibody (Sigma Inc., USA) was used for the GFP tagged-Rac1 detection. The antibody used to recognize the loading control tubulin was rat anti-tubulin (Bio-Rad Inc., USA). The secondary antibodies were IRye800CW goat anti-mouse IgG (H+L) conjugate (Li-Cor Biosciences) (1:10,000), IRye800CW goat anti-rabbit IgG (H+L) conjugate (Li-Cor Biosciences) (1:10,000), and IRye600RD goat anti-rat IgG(H+L) conjugate (Li-Cor Biosciences) (1:10,000). Densitometry quantification of active GTP-bound GTPases versus the total GTPase input bands was performed with ImageJ (National Institutes of Health, Bethesda, MD) from images generated on an Odyssey Imager (Li-Cor Biosciences).

### Immunofluorescent imaging of β (1,3)-glucan exposure.

To stain the *lrg1ΔΔ* mutant, *Candida* cells were grown overnight in YPD medium at 30°C. The culture was collected and processed for immunostaining. The staining protocol was followed as described previously (17).

### Flow cytometry.

To stain the *STE11^ΔN467^* strain under the regulation of *P_tetOFF_* promoter over time, overnight cultures in YPD medium with doxycycline were diluted to an OD_600_ of 0.2, and cells were collected after growth in fresh YPD medium without doxycycline at 1-h, 2-h, 4-h, and 6-h time points. To stain the *lrg1ΔΔ* mutant, an overnight culture in YPD was used. The staining protocol and gating strategy for all of these samples were followed as described previously (17). Flow cytometry data were obtained for 100,000 gated events per strain, experiments were performed in triplicate, and the data were analyzed using the FlowJo software package (version 10.11; FlowJo LLC, OR, USA).

### Enzyme-linked immunosorbent assay (ELISA) of TNF-α.

To activate Ste11^ΔN467^ expression under the *P_tetOFF_* regulation, the *P_tetOFF_-STE11^ΔN467^* mutant strain was grown in YPD without doxycycline overnight. Otherwise, strains were grown with doxycycline overnight. RAW264.7 murine macrophages were in used in this assay. The manufacturer’s instructions for an ELISA kit (R&D Systems, USA) were followed. Each sample was represented by three individual replicates, and the statistical analysis was performed by using two-way analysis of variance (ANOVA) (GraphPad Prism, v7.04 software).

### Mouse model.

Outbred male ICR mice were used in this study. C. albicans wild-type strain DAY286 and the *P_tetOFF_*-*STE11^ΔN467^* strain were cultured overnight at 30°C in 50 ml of YPD medium with 0.5 μg/ml of doxycycline to repress Ste11^ΔN467^ expression. The overnight culture was counted via hemocytometer and diluted to 10^6^ cells/ml. The diluted fungal cells were plated on YPD media to test for viability. Mice were injected via the lateral tail vein with 0.1 ml of the fungal cell suspension. Mice were given either drinking water supplemented with 2 mg/ml doxycycline plus 5% sucrose to cover the bitter taste of the antibiotics (67) or 5% sucrose water alone as a control. Mice were monitored closely for 21 days for signs of illness. For the fungal burden counting experiment, mice were sacrificed 4 days postinfection. Kidneys were harvested, homogenized, serially diluted in water, and plated on YPD media. The plates were incubated at 30°C for 2 days to determine CFU per gram of kidney.

### Ethics statement.

All mouse model experiments in this study were performed under an animal protocol (1083) that was approved by the University of Tennessee Institutional Animal Care and Use Committee (IACUC), and we followed the ethical guidelines set forth by the National Institutes of Health (NIH) for the ethical treatment of animals.

### RNA extraction.

The RNA extraction protocol was modified from a protocol described previously (68). A 15-ml volume of yeast overnight culture were collected and washed 3 times with phosphate-buffered saline (PBS). A 750-μl volume of TES buffer (Tris-Cl [10 mM, pH 7.6], EDTA [10 mM], sodium dodecyl sulfate [SDS; 0.5% {wt/vol}]), prepared with RNase-free water (RPI Corp., USA) and 750 μl of acid phenol (Thermo Fisher, USA) (pH 4.5), was added to the pellet, and an equal volume of 150-to-212-μm-diameter acid-washed glass beads (Millipore-Sigma, USA) was added to each tube. Cells were mechanically disrupted in a Biospec Mini-BeadBeater (BioSpec Products Inc., USA) with 4 rounds of 1 min homogenization at 4°C and 2-min intervals for each cycle on ice. Samples were placed in a 65°C heat block for 30 min and subjected to thorough vortex mixing every 10 min. The mixture was centrifuged for 5 min at 13,000 rpm at room temperature, and the aqueous layer was transferred to a fresh tube containing 700 μl of acid phenol (pH 4.5) and spun for 5 min. The aqueous layer was transferred and washed twice with 600 μl of neutral pH phenol (Thermo Fisher, USA), followed by washing with 600 μl of chloroform until the interface was clean. The supernatant was further transferred to the tube containing 150 μl of 3 M sodium acetate, followed by addition of 1 ml of 100% ethanol, and the mixture was placed in a freezer and maintained at –80°C overnight to precipitate the nucleic acid. The pellet was collected by centrifugation at 13,000 rpm at 4°C for 10 min and further washed with 500 μl of ice-cold 70% ethanol. The pellet was dried at room temperature and resuspended in 100 μl of RNase-free water. DNA was removed by using a Turbo DNA-free kit (Thermo Fisher, USA) following the manufacturer’s instructions.

RNA samples were analyzed for quality by the use of a 2100 series bioanalyzer (Agilent Technologies, USA) at the University of Tennessee Genomics Core, followed by cDNA library preparation and sequencing. Barcoded cDNA libraries were prepared with an Illumina TruSeq stranded mRNA sample preparation kit according to the specifications of the manufacturer (Illumina, Inc., USA). The quality of the libraries was validated by the use of a bioanalyzer (Agilent Technologies, USA), and the contents were then arranged on a flow cell using MiSeq reagent kit v3 (Illumina, Inc., USA) (150 cycles) and sequenced on an Illumina MiSeq M04398 machine.

### Bioinformatic analysis.

All the operations were performed with CLC Genomics Workbench v.12.0 software (Qiagen, Germany). Trimming was performed with the quality score limit set at 0.01 and ambiguity set at 0. The read length to be discarded was set to below 50. The C. albicans SC5314 reference genome and corresponding annotations (version A22-s07-m01-r71) were downloaded from the Candida Genome Database and were converted to a haploid genome and annotation set (A alleles) to avoid issues with mapping specificity (69). The genome was annotated with the GFF file plugin available on CLC Genomics Workbench v.12.0 software. The mapping parameters used were as follows: mismatch cost = 2, insertion cost = 3, deletion cost =3, length fraction = 0.8, and similarity fraction = 0.8. Expression values for each gene were calculated from unique gene reads (the maximum number of hits for a read was set to 1) and normalized by gene length and sequencing depth, yielding the expression value of transcripts per million (TPM). To determine if gene expression values were correct, the TPM reads of five housekeeping genes (*TEF1*, *ACT1*, *TUB1*, *ENO1*, and *PMA1*) were evaluated to calculate the average for each condition, which was further plotted as described previously (70). To identify genes that were differentially expressed under treatment and control conditions, the *P* values calculated for the individual genes were adjusted for false-discovery rate (FDR), and genes with an FDR-adjusted *P* value of *<*0.05 in the treatment group were considered differentially expressed. Expression values exceeding a factor of 2 (fold change [either higher or lower than that calculated for the control]) were considered to represent significantly differential expression. The RNA sequence reads have been deposited in the NCBI Sequence Read Archive under the BioProject number PRJNA559867.
