# Supplementary figures and images for: Lrg1 Regulates β (1,3)-Glucan Masking in Candida albicans through the Cek1 MAP Kinase Pathway
Source: mBio. 2019 Sep 17;10(5):e01767-19. doi: 10.1128/mBio.01767-19 (PMC6751057; doi:10.1128/mBio.01767-19)

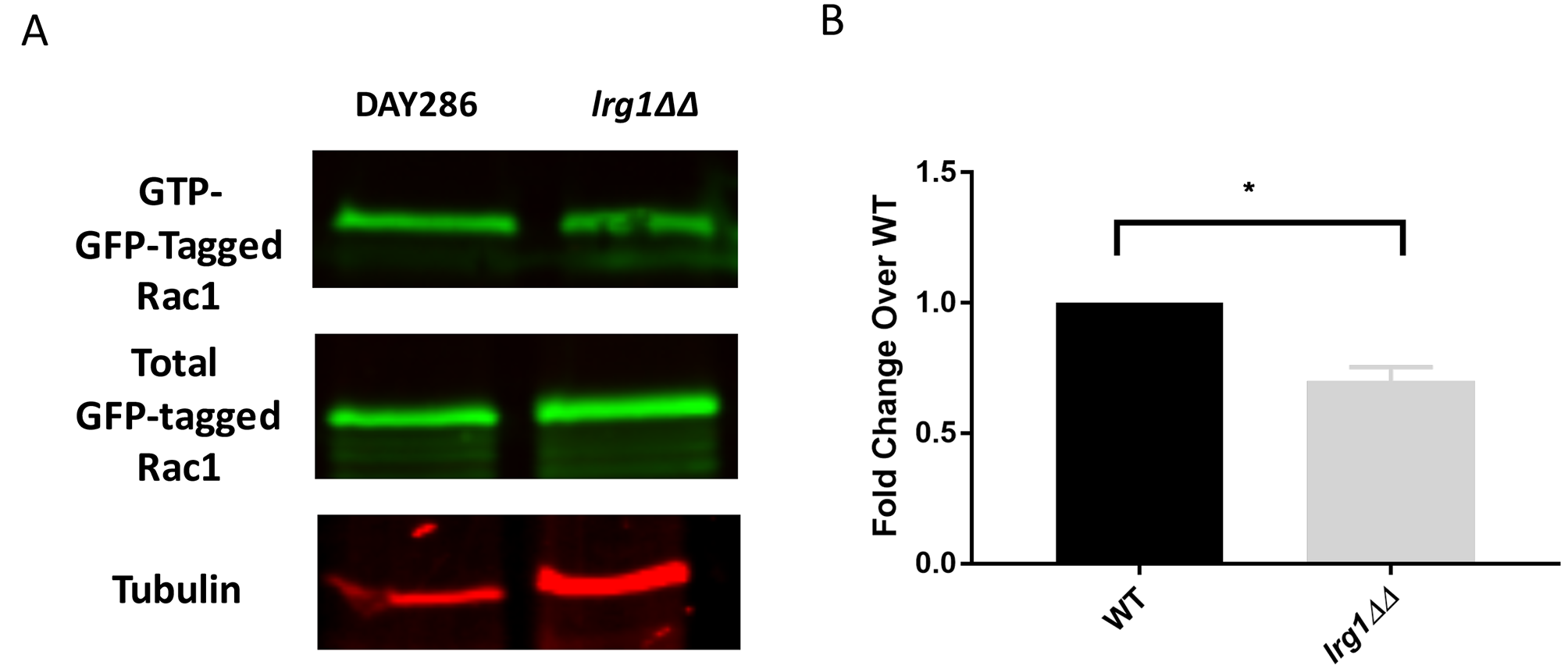

Supplement: FIG S1 [file mBio.01767-19-sf001.tif]

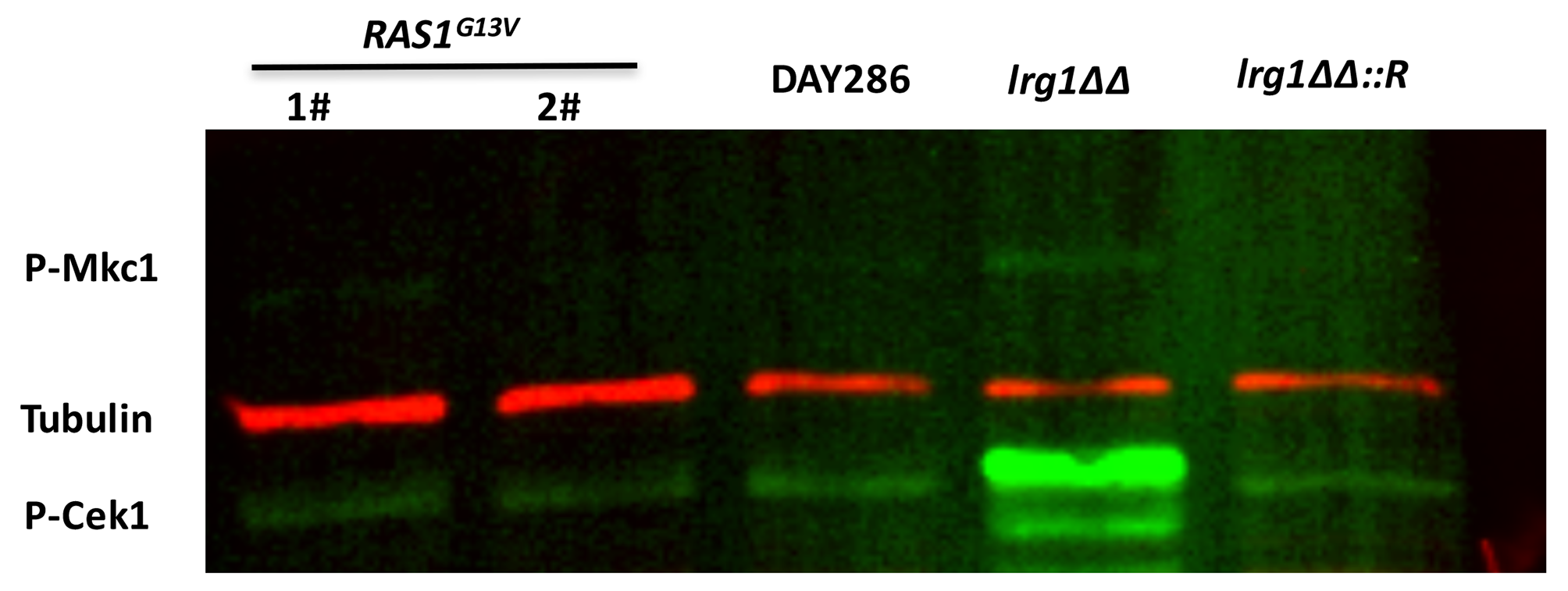

Supplement: FIG S2 [file mBio.01767-19-sf002.tif]

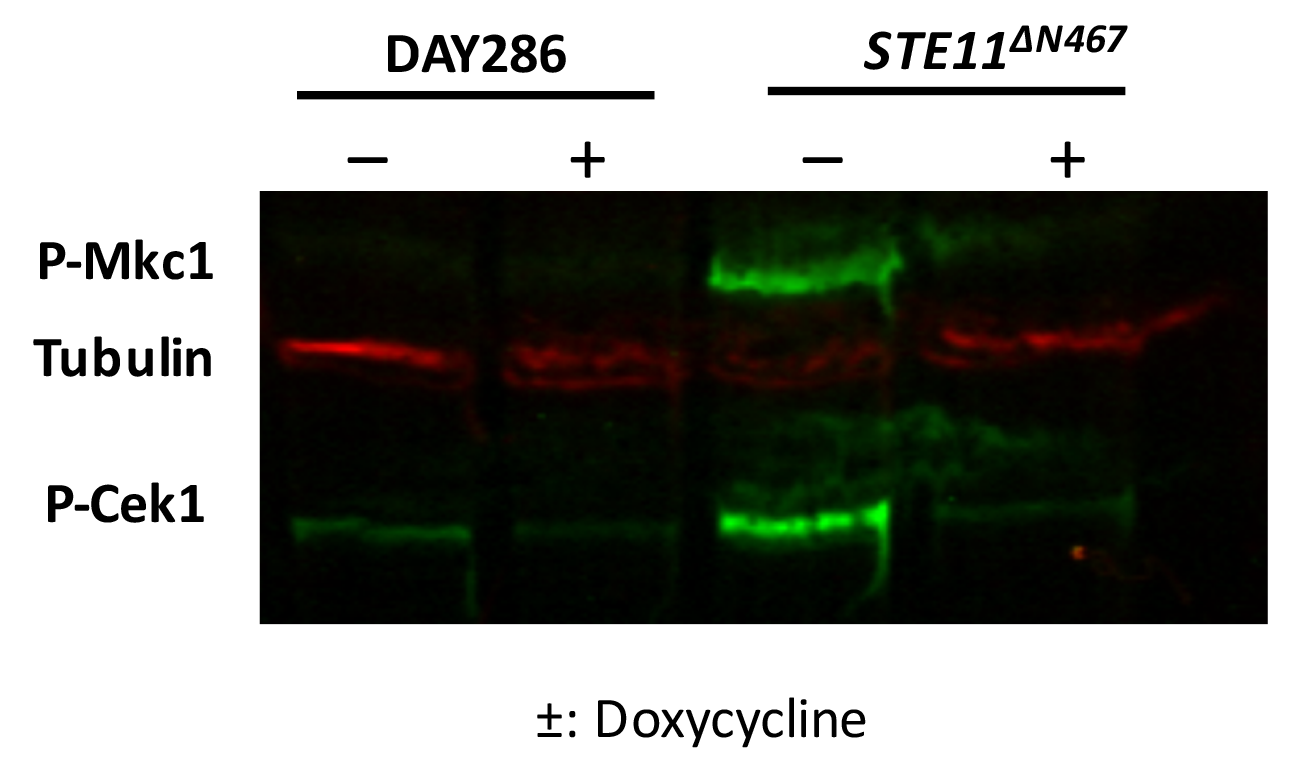

Supplement: FIG S3 [file mBio.01767-19-sf003.tif]

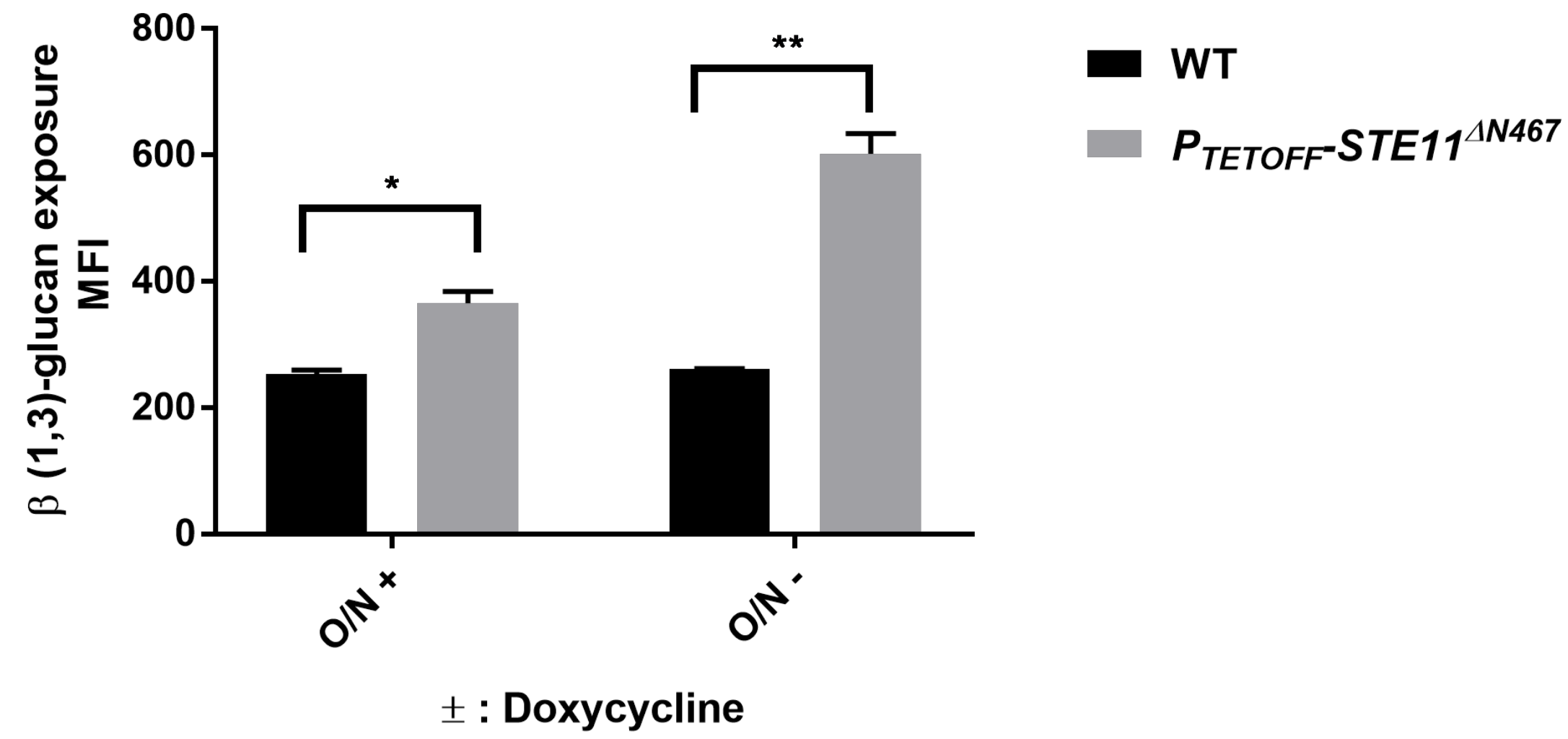

Supplement: FIG S4 [file mBio.01767-19-sf004.tif]

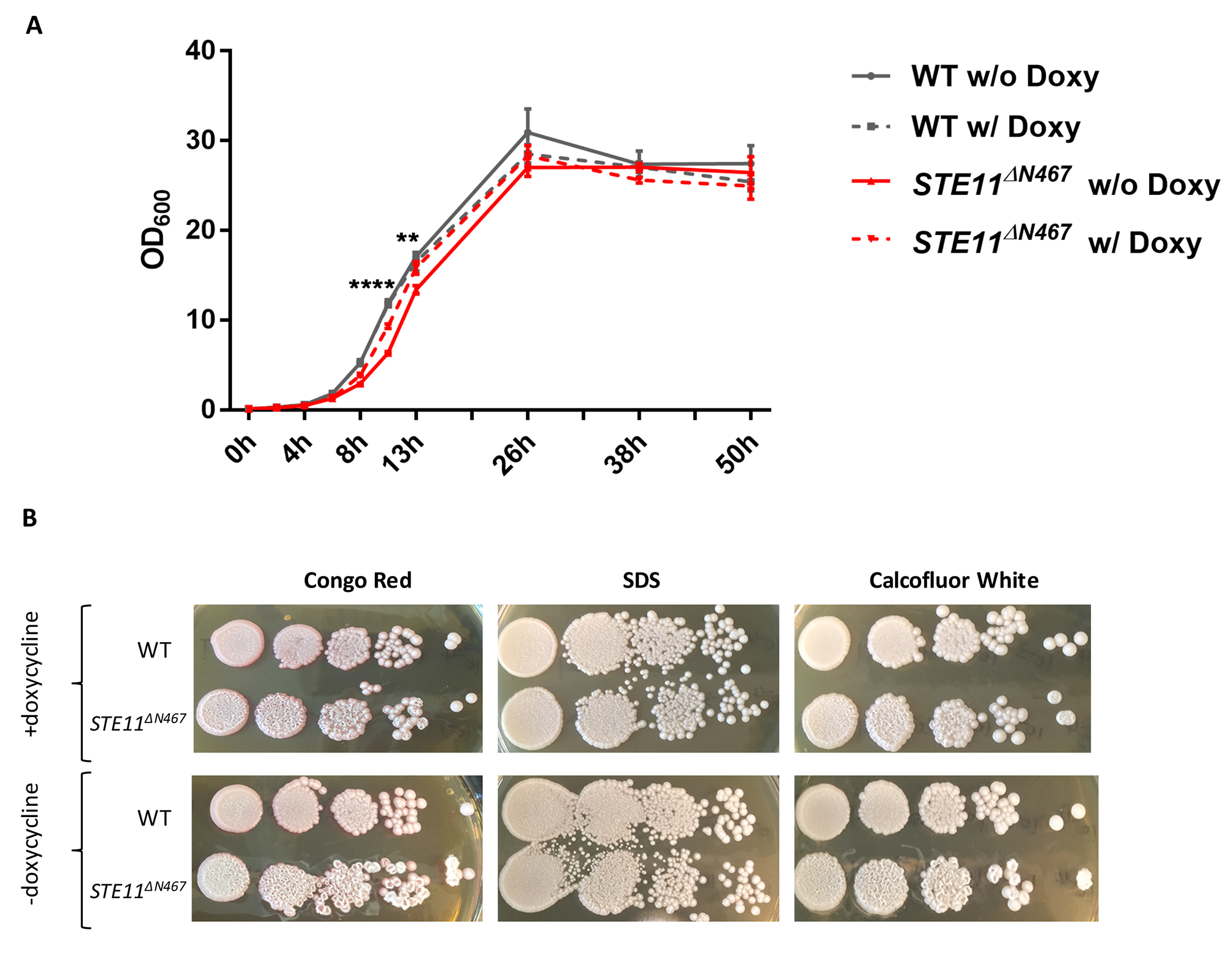

Supplement: FIG S5 [file mBio.01767-19-sf005.tif]

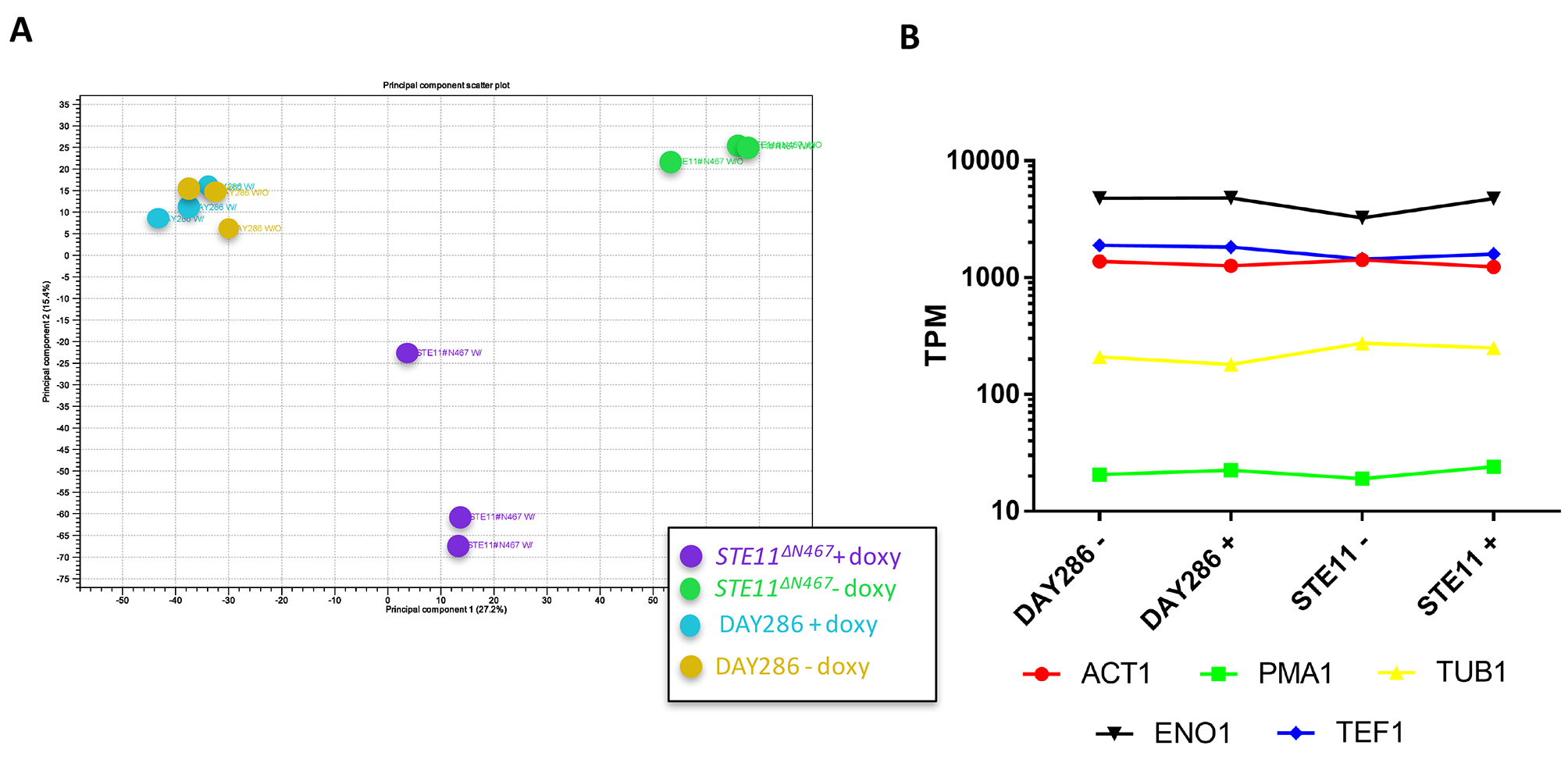

Supplement: FIG S6 [file mBio.01767-19-sf006.tif]

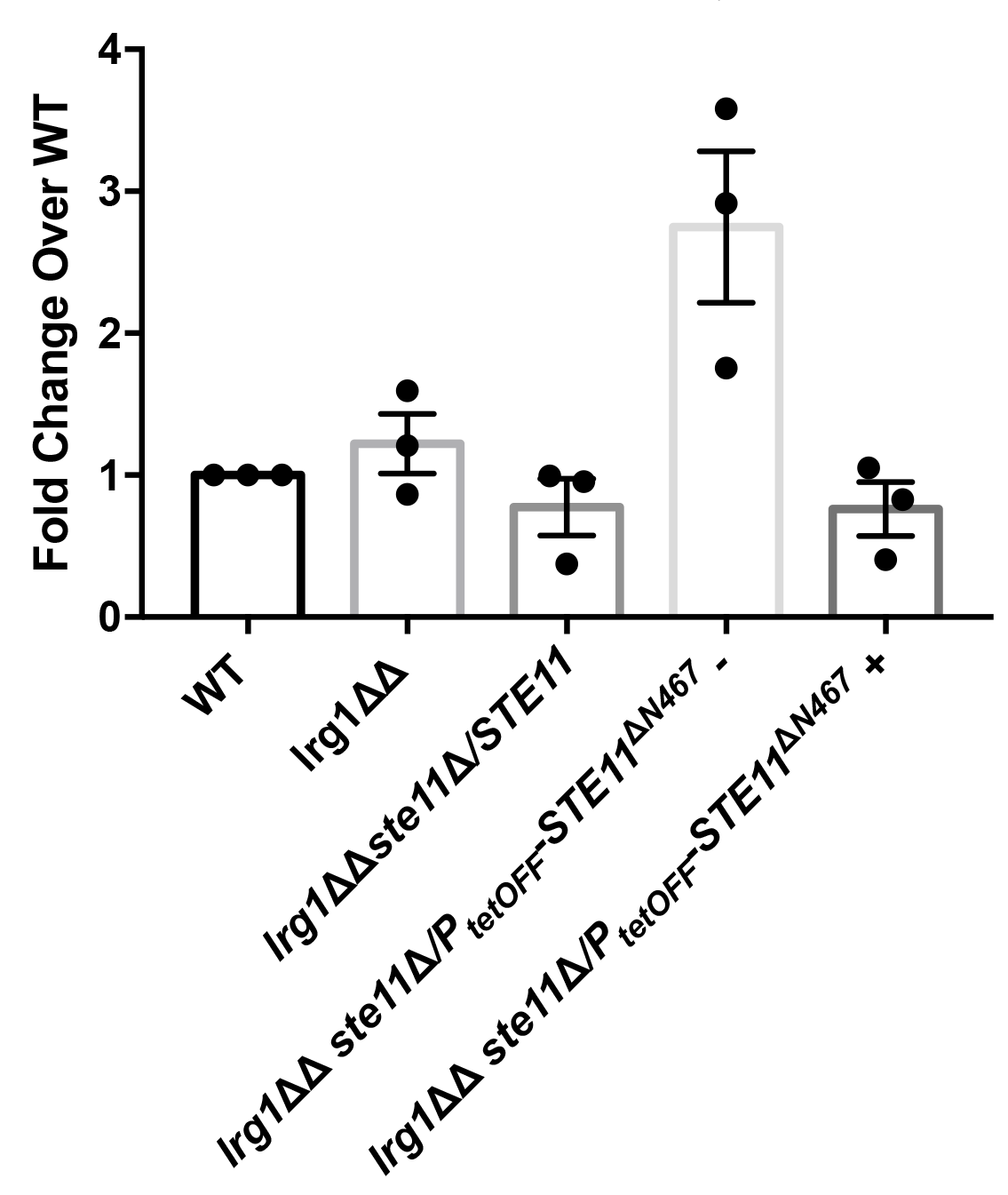

Supplement: FIG S7 [file mBio.01767-19-sf007.tif]
