# Supplementary material for: Lrg1 Regulates β (1,3)-Glucan Masking in Candida albicans through the Cek1 MAP Kinase Pathway
Source: mBio. 2019 Sep 17;10(5):e01767-19. doi: 10.1128/mBio.01767-19 (PMC6751057; doi:10.1128/mBio.01767-19)
Supplement: TABLE S1 [file mBio.01767-19-st001.docx]

| **Table S1. Genes differentially expressed when *STE11^∆N467^* is overexpressed.** | | | | |
| --- | --- | --- | --- | --- |
| **Gene Name** | **Function** | Fold Change | | |
|  |  | vs. WT.  –doxy | vs. WT +doxy | vs. *STE11^ΔN467^*+doxy |
| PBR1 | Protein of unknown function; required for cohesion, adhesion, and RPMI biofilm formation; induced by alpha pheromone in white cells; fluconazole-induced; Spider biofilm induced | 1,269.77 | 1,276.88 | 333.46 |
| PGA13 | GPI-anchored cell wall protein involved in cell wall synthesis; required for normal cell surface properties; induced in oralpharyngeal candidasis; Spider biofilm induced; Bcr1-repressed in RPMI a/a biofilms | 114.57 | 93.25 | 22.48 |
| *C2_09880C_A* | Putative protein of unknown function; Plc1-regulated; transcript induced by Mnl1 under weak acid stress; flow model, rat catheter, Spider biofilm induced | 59.07 | 32.66 | 14.68 |
| XOG1 | Exo-1,3-beta-glucanase; 5 glycosyl hydrolase family member; affects sensitivity to chitin and glucan synthesis inhibitors; not required for yeast-to-hypha transition or for virulence in mice; Hap43-induced; Spider biofilm induced | 51.34 | 55.89 | 37.56 |
| *C2_02220C_A* | Protein of unknown function; F-12/CO2 early biofilm induced | 47.72 | 46.24 | 18.21 |
| RBT4 | Pry family protein; required for virulence in mouse systemic/rabbit corneal infections; not filamentation; mRNA binds She3, is localized to hyphal tips; Hap43-induced; in both yeast and hyphal culture supernatants; Spider biofilm induced | 40.37 | 31.85 | 43.55 |
| *C7_02280W_A* | Ortholog of C. parapsilosis CDC317 : CPAR2_808370, C. dubliniensis CD36 : Cd36_72070, Candida orthopsilosis Co 90-125 : CORT_0C00800 and Candida albicans WO-1 : CAWG_05577 | 39.92 | 43.07 | 19.01 |
| PGA31 | Cell wall protein; putative GPI anchor; expression regulated upon white-opaque switch; induced by Congo Red and cell wall regeneration; Bcr1-repressed in RPMI a/a biofilms | 36.75 | 44.22 | 17.82 |
| SAP7 | Pepstatin A-insensitive secreted aspartyl protease; self-processing; expressed in human oral infection; Ssn6p-regulated; role in murine intravenous infection; induced during, but not required for, murine vaginal infection; N-glycosylated | 36.55 | 16.76 | 14.56 |
| KRE1 | Cell wall glycoprotein; beta glucan synthesis; increases glucan content in S. cerevisiae kre1, complements killer toxin sensitivity; caspofungin induced; Spider/rat catheter/flow model biofilm induced; Bcr1-repressed in RPMI a/a biofilms | 34.49 | 25.71 | 18.03 |
| *C7_02260W_A* | Ortholog of C. parapsilosis CDC317 : CPAR2_808350, C. dubliniensis CD36 : Cd36_72060, Candida orthopsilosis Co 90-125 : CORT_0C00820 and Candida albicans WO-1 : CAWG_05575 | 33.04 | 31.96 | 17.09 |
| DAG7 | Secretory protein; a-specific, alpha-factor induced; mutation confers hypersensitivity to toxic ergosterol analog; fluconazole-induced; induced during chlamydospore formation in C. albicans and C. dubliniensis | 27.47 | 28.28 | 24.06 |
| FAV1 | Protein with weak similarity to S. cerevisiae Fus2p; induced by alpha pheromone mating factor in MTLa/MTLa opaque cells | 23.68 | 36.82 | 8.76 |
| RBR1 | Glycosylphosphatidylinositol (GPI)-anchored cell wall protein; required for filamentous growth at acidic pH; expression repressed by Rim101 and activated by Nrg1; Hap43-induced | 21.41 | 28.04 | 5.53 |
| LDG3 | Putative LDG family protein; F-12/CO2 early biofilm induced | 20.45 | 23.8 | 11.02 |
| *C6_02100W_A* | Secreted potein; Hap43-repressed; fluconazole-induced; regulated by Tsa1, Tsa1B under H2O2 stress conditions; induced by Mnl1p under weak acid stress; Spider biofilm induced | 18.17 | 20.69 | 8.74 |
| *C1_07040C_A* | Pry family pathogenesis-related protein; oral infection upregulated gene; mutant has reduced capacity to damage oral epithelial cells | 16.15 | 13.23 | 13.55 |
| MRV8 | Protein of unknown function; Spider biofilm induced | 15.68 | 15.16 | 11.93 |
| PGA23 | Putative GPI-anchored protein of unknown function; Rim101-repressed; Cyr1-regulated; colony morphology-related gene regulation by Ssn6 | 15.46 | 17.02 | 12.49 |
| *CR_05330W_A* | Protein of unknown function; Spider biofilm repressed | 13.1 | 13 | 7.97 |
| *C1_05970W_A* | Putative adhesin-like protein; macrophage-induced gene | 13.06 | 14.56 | 14.35 |
| HWP1 | Hyphal cell wall protein; host transglutaminase substrate; opaque-, a-specific, alpha-factor induced; at MTLa side of conjugation tube; virulence complicated by URA3 effects; Bcr1-repressed in RPMI a/a biofilms; Spider biofilm induced | 13.05 | 6.29 | 4.22 |
| IFF11 | Secreted protein required for normal cell wall structure and for virulence; member of the IFF family; Hap43p-repressed gene | 12.7 | 17.03 | 9.93 |
| *C4_04380C_A* | Protein of unknown function; Spider biofilm induced | 12.04 | 12.56 | 11.25 |
| FAV2 | Adhesin-like protein; induced by mating factor in MTLa/a opaque cells, in cyr1 null, in filaments; regulated by Nrg1, Rfg1, Tup1, Tec1, Efg1, Ntd80, Rob1, Brg1; induced in oralpharyngeal candidasis; Spider biofilm induced | 10.33 | 7.52 | 10.97 |
| *C4_03500C_A* | Protein of unknown function; regulated by Tsa1, Tsa1B in minimal media at 37 degrees C | 9.13 | 8.58 | 36.84 |
| CEK1 | ERK-family protein kinase; required for wild-type yeast-hypha switch, mating efficiency, virulence in mice; Cst20-Hst7-Cek1-Cph1 MAPK pathway regulates mating, and invasive hyphal growth under some conditions; Spider biofilm induced | 9.06 | 6.85 | 7.45 |
| KCH1 | Ortholog of Kch1 a potassium transporter; mediates K+ influx and activates high-affinity Ca2+ influx system during mating pheromone response in S. cerevisiae; induced by alpha pheromone in SpiderM medium | 8.95 | 12.59 | 9.23 |
| ALK8 | Alkane-inducible cytochrome P450; catalyzes hydroxylation of lauric acid to hydroxylauric acid; overproduction causes fluconazole resistance in WT and causes multidrug resistance in a cdr1 cdr2 double mutant; rat catheter biofilm repressed | 8.84 | 7.53 | 4.37 |
| CPH1 | Transcription factor; for mating, filamentation on solid media, pheromone-stimulated biofilms; in pathway with Ess1, Czf1; required with Efg1 for host cytokine response; regulates galactose metabolism genes; rat cathether biofilm repressed | 8.46 | 9.24 | 7.52 |
| *C5_03440W_A* | Protein of unknown function | 7.98 | 8.3 | 6.92 |
| CPP1 | VH1 family MAPK phosphatase; regulates Cst20-Hst7-Cek1-Cph1 filamentation pathway; negatively regulates mating, represses yeast-hyphal switch; required for virulence in mice; yeast-enriched; induced by alpha pheromone in SpiderM medium; | 6.7 | 8.03 | 6.9 |
| CSH1 | Aldo-keto reductase; role in fibronectin adhesion, cell surface hydrophobicity; regulated by temperature, growth phase, benomyl, macrophage interaction; azole resistance associated; Spider biofilm induced; rat catheter biofilm repressed | 6.61 | 8.4 | 5.01 |
| *C5_04940W_A* | Maltase; induced during growth on sucrose; induced by alpha pheromone in SpiderM medium; early-stage flow model biofilm indced | 6.57 | 6.29 | 5.56 |
| STE11 | Protein similar to S. cerevisiae Ste11p; mutants are sensitive to growth on H2O2 medium | 6.56 | 6.02 | 8.53 |
| *C4_01330W_A* | Protein of unknown function; Spider biofilm induced | 6.45 | 4.56 | 2.52 |
| PRM1 | Putative membrane protein with a predicted role in membrane fusion during mating; Hap43p-repressed gene; protein induced during the mating process | 6.16 | 5.83 | 7.85 |
| *CR_09930W_A* | Protein of unknown function; induced by alpha pheromone in SpiderM medium | 5.99 | 5.11 | 4.88 |
| *C6_04190C_A* | Protein of unknown function; Spider biofilm induced | 5.92 | 6.05 | 3.84 |
| *CR_04770C_A* | Ortholog of C. dubliniensis CD36 : Cd36_30140, C. parapsilosis CDC317 : CPAR2_204040, Candida tenuis NRRL Y-1498 : CANTEDRAFT_114703 and Debaryomyces hansenii CBS767 : DEHA2G22880g | 5.83 | 6.28 | 2.89 |
| PGA30 | GPI-anchored protein of cell wall | 5.81 | 19.39 | 3.78 |
| PTR2 | Oligopeptide transporter involved in uptake of di-/tripeptides; highly induced during chlamydospore formation in both C. albicans and C. dubliniensis | 5.73 | 5.02 | 2.27 |
| GFA1 | Glucosamine-6-phosphate synthase, homotetrameric enzyme of chitin/hexosamine biosynthesis; inhibited by UDP-GlcNAc, FMDP, N-acyl peptide, kanosamine-6-P; functional homolog of S. cerevisiae Gfa1p; Cagrowth-phase regulated; catalytic Cys | 5.65 | 7.04 | 4.98 |
| GST3 | Glutathione S-transferase; expression regulated upon white-opaque switch; induced by human neutrophils; peroxide-induced; induced by alpha pheromone in SpiderM medium; Spider biofilm induced | 5.52 | 5.73 | 2.45 |
| *C4_02260C_A* | Protein of unknown function; repressed by prostaglandins; Hap43-induced, Spider biofilm induced | 5.48 | 17.53 | 6.59 |
| CRH11 | GPI-anchored cell wall transglycosylase, putative ortholog of S. cerevisiae Crh1p; predicted glycosyl hydrolase domain; similar to Csf4p and to antigenic A. fumigatus Aspf9; predicted Kex2p substrate; caspofungin-induced | 4.95 | 4.45 | 3.31 |
| WSC2 | Putative cell wall integrity and stress response protein; mRNA binds She3; Spider biofilm induced | 4.74 | 5.31 | 2.99 |
| PGA58 | Putative GPI-anchored protein; transcription is positively regulated by Tbf1p | 4.59 | 4.42 | 2.97 |
| MNN12 | Predicted alpha-1,3-mannosyltransferase activity with a role in protein glycosylation | 4.25 | 4.88 | 3.61 |
| *C1_09430W_A* | Putative membrane protein; induced by alpha pheromone in SpiderM medium; Hap4-induced gene; Spider biofilm induced | 4.24 | 5.89 | 4.82 |
| *C1_03870C_A* | Predicted heme-binding stress-related protein; Tn mutation affects filamentous growth; induced during chlamydospore formation in C. albicans and C. dubliniensis; Spider biofilm induced | 4.23 | 4.46 | 4.99 |
| *C6_00810C_A* | Protein of unknown function; Hap43-repressed gene | 4.21 | 2.98 | 3.56 |
| KAR4 | Transcription factor; required for gene regulation in response to pheromones; ortholog of S. cerevisiae Kar4; role in karyogamy; opaque-specific, a-specific; induced by alpha factor | 4.21 | 3.62 | 2.99 |
| *CR_00090C_A* | Protein of unknown function; stationary phase enriched protein; induced upon yeast-hypha transition; benomyl or caspofungin induced; Hap43-repressed; Spider biofilm induced | 4.17 | 3.76 | 2.01 |
| DDR48 | Immunogenic stress-associated protein; filamentation regulated; induced by benomyl/caspofungin/ketoconazole or in azole-resistant strain; Hog1, farnesol, alkaline repressed; stationary phase enriched; Spider, flow model biofilm induced | 4.16 | 4.62 | 3.57 |
| OPY2 | Predicted transmembrane protein; role in cell wall biogenesis; required for Cek1 phosphorylation; Spider biofilm induced | 4.14 | 4.11 | 2.81 |
| MNN14 | Predicted alpha-1,3-mannosyltransferase activity with a role in protein glycosylation; Hap43-repressed; Spider biofilm induced | 4 | 3.91 | 2.57 |
| *C2_05040C_A* | Ortholog(s) have cellular bud neck, fungal-type vacuole localization | 3.94 | 3.83 | 3.47 |
| *C6_04420W_A* | Protein of unknown function; GlcNAc-induced protein; Spider biofilm induced; rat catheter biofilm repressed | 3.94 | 2.64 | 2.84 |
| CHS8 | Chitin synthase required for synthesis of long-chitin fibrils; nonessential; 8 or 9 membrane spanning regions; mRNA present in yeast and hyphae; induced during cell wall regeneration; flow model biofilm repressed | 3.65 | 3.74 | 2.66 |
| *C1_11730W_A* | Protein with SEL-1 like protein domain; early-stage flow model biofilm induced | 3.64 | 3.63 | 2.81 |
| CHS2 | Chitin synthase; nonessential; required for wild-type chitin deposition in hyphae; transcript regulated during dimorphic transition; Chs1 and Chs2, but not Chs3, are inhibited by the protoberberine HWY-289; flow model biofilm repressed | 3.56 | 4.08 | 3.26 |
| PGA54 | GPI-anchored protein; Hog1-repressed; induced in cyr1 or efg1 mutant or in hyphae; colony morphology-related gene regulation by Ssn6; induced in RHE model; mRNA binds She3; regulated in Spider biofilms by Tec1, Egf1, Ntd80, Rob1, Brg1 | 3.47 | 3.69 | 3.42 |
| GPX1 | Putative thiol peroxidase; rat catheter and Spider biofilm induced | 3.42 | 3.75 | 2.03 |
| *C7_02250W_A* | Ortholog of C. dubliniensis CD36 : Cd36_72050, C. parapsilosis CDC317 : CPAR2_301140, Candida tenuis NRRL Y-1498 : CANTEDRAFT_135055 and Debaryomyces hansenii CBS767 : DEHA2E07678g | 3.35 | 4.17 | 2.87 |
| *C1_07990C_A* | Similar to cell-wall mannoproteins; induced in low iron; induced in cyr1 homozygous null; regulated by osmotic and oxidative stress via Hog1; Spider biofilm induced | 3.25 | 3.7 | 3.17 |
| *C2_10150W_A* | Secreted protein; fluconazole-induced | 3.1 | 2.84 | 2.92 |
| *CR_07850W_A* | Ortholog of C. dubliniensis CD36 : Cd36_33530, C. parapsilosis CDC317 : CPAR2_201980, Candida tenuis NRRL Y-1498 : CANTEDRAFT_134293 and Debaryomyces hansenii CBS767 : DEHA2A10164g | 3.1 | 2.65 | 2.03 |
| RLM1 | Putative transcription factor; required for wild-type resistance to cell wall perturbation, caspofungin treatment; regulates caspofungin induction of PGA13 | 3.07 | 3.23 | 2.31 |
| UME7 | Putative transcription factor with zinc cluster DNA-binding motif; similar to S. cerevisiae Ume6p, which is a transcription factor involved in the regulation of meiotic genes | 3.02 | 3.23 | 2.51 |
| *C1_04010C_A* | Protein with a NADP-dependent oxidoreductase domain; transcript induced by ketoconazole; rat catheter and Spider biofilm induced | 2.99 | 3.89 | 2.27 |
| BUD5 | Predicted GTP/GDP exchange factor for Rsr1; rat catheter biofilm induced | 2.91 | 3.18 | 2.1 |
| UAP1 | UDP-N-acetylglucosamine pyrophosphorylase, catalyzes biosynthesis of UDP-N-acetylglucosamine from UTP and N-acetylglucosamine 1-phosphate; functional homolog of S. cerevisiae Qri1p; alkaline upregulated | 2.9 | 4.31 | 3.82 |
| BRG1 | Transcription factor; recruits Hda1 to hypha-specific promoters; Tn mutation affects filamentation; Hap43-repressed; Spider and flow model biofilm induced; required for Spider biofilm formation; Bcr1-repressed in RPMI a/a biofilms | 2.85 | 4.05 | 2.76 |
| AXL2 | Ortholog of S. cerevisiae Axl2; a plasma membrane protein involved in determination of budding pattern; O-glycosylated by Pmt4; mutant is viable | 2.8 | 4.24 | 3.54 |
| YKE2 | Possible heterohexameric Gim/prefoldin protein complex subunit; role in folding alpha-tubulin, beta-tubulin, and actin; transcript induced by yeast-to-hypha switch; regulated by Nrg1, Tup1; Spider and flow model biofilm induced | 2.78 | 3.28 | 2.18 |
| *C6_02210W_A* | Protein of unknown function; oxidative stress-induced via Cap1; induced by alpha pheromone in SpiderM medium | 2.67 | 2.4 | 2.21 |
| CHS7 | Protein required for wild-type chitin synthase III activity; similar to (but not functional homolog of) S. cerevisiae Chs7p, which effects ER export of Chs3p; induced cyr1 mutant hyphae and ras1 yeast-form cells; Spider biofilm induced | 2.58 | 2.6 | 2.68 |
| RGA2 | Putative GTPase-activating protein (GAP) for Rho-type GTPase Cdc42; involved in cell signaling pathways controlling cell polarity; induced by low-level peroxide stress; flow model biofilm induced | 2.54 | 2.55 | 2.4 |
| MRV3 | Ortholog of Candida albicans WO-1 : CAWG_04793 | 2.54 | 2.42 | 2.08 |
| *C2_07790C_A* | Protein of unknown function; induced by alpha pheromone in SpiderM medium | 2.53 | 3.05 | 2.97 |
| *C7_01940C_A* | Pheromone-regulated protein (Prm10) of S. cerevisiae; colony morphology-related gene regulation by Ssn6; induced by Mnl1 under weak acid stress; possibly essential gene, disruptants not obtained by UAU1 method; Spider biofilm induced | 2.53 | 2.37 | 2.26 |
| PMS1 | Putative DNA mismatch repair factor; ortholog of S. cerevisiae PMS1 which is an ATP-binding protein involved in DNA mismatch repair | 2.5 | 3.33 | 2.94 |
| *CR_09580C_A* | Ortholog of S. cerevisiae Pba1 that is involved in 20S proteasome assembly; upregulated in a cyr1 null mutant; contains a 5' UTR intron | 2.48 | 3.03 | 2.33 |
| *C1_05440C_A* | Protein of unknown function; flow model biofilm induced; Spider biofilm induced | 2.45 | 2.14 | 2.01 |
| DFI1 | Cell-surface associated glycoprotein; promotes activation of Cek1 in a matrix-dependent manner; N-glycosylated; Spider biofilm induced | 2.43 | 2.63 | 2.28 |
| *CR_09090C_A* | Ortholog of C. dubliniensis CD36 : Cd36_34510, Candida tropicalis MYA-3404 : CTRG_05938 and Candida albicans WO-1 : CAWG_02183 | 2.36 | 2.92 | 2.01 |
| *C4_01420W_A* | Ortholog of C. dubliniensis CD36 : Cd36_41430, Candida tropicalis MYA-3404 : CTRG_00187 and Candida albicans WO-1 : CAWG_03642 | 2.3 | 2.59 | 2.15 |
| HGC1 | Hypha-specific G1 cyclin-related protein involved in regulation of morphogenesis, biofilm formation; Cdc28-Hgc1 maintains Cdc11 S394 phosphorylation during hyphal growth; required for virulence in mice; regulated by Nrg1, Tup1, farnesol | 2.29 | 3.29 | 2.56 |
| *C3_02290W_A* | Protein similar to S. cerevisiae Ydr282cp; transposon mutation affects filamentous growth; Hap43p-repressed gene | 2.25 | 2.18 | 2.61 |
| KRE6 | Essential beta-1,6-glucan synthase subunit; change in mRNA length, not abundance, at yeast-hypha transition; alkaline induced by Rim101, on cell wall regeneration; Spider biofilm induced; Bcr1-repressed in RPMI a/a biofilms | 2.24 | 2.7 | 2.34 |
| HAC1 | bZIP transcription factor; role in unfolded protein response and control of morphology; transcript undergoes atypical splicing at C terminus under ER stress; induced during mating and by caspofungin; mRNA binds She3; Spider biofilm induced | 2.12 | 2.32 | 2.59 |
| PTP2 | Predicted protein tyrosine phosphatase; involved in regulation of MAP kinase Hog1 activity; induced by Mnl1 under weak acid stress; rat catheter and Spider biofilm induced | 2.11 | 2.22 | 2.45 |
| ENG1 | Endo-1,3-beta-glucanase; ortholog of S. cerevisiae Dse4 needed for cell separation; caspofungin, fluconazole repressed; repressed by alpha pheromone in SpiderM medium; flow model biofilm induced; rat catheter biofilm repressed | -2.09 | -2.14 | -2.79 |
| *C7_01170C_A* | Putative oxidoreductase; mutation confers hypersensitivity to toxic ergosterol analog; rat catheter and Spider biofilm induced | -2.17 | -4.89 | -4.56 |
| *C1_11990W_A* | Putative cell wall adhesin-like protein; repressed in core caspofungin response and by alpha pheromone in SpiderM medium; transcript reduced in ace2 mutant; flow model, rat catheter and Spider biofilm repressed | -2.22 | -2.06 | -3.17 |
| *C2_06800C_A* | Protein of unknown function; Spider biofilm induced | -2.28 | -2.94 | -2.31 |
| TNA1 | Putative nicotinic acid transporter; detected at germ tube plasma membrane by mass spectrometry; transcript induced upon phagocytosis by macrophage; rat catheter biofilm induced | -2.29 | -2.93 | -4.18 |
| *C4_02080W_A* | Protein with a mitochondrial carrier protein domain; possibly an essential gene, disruptants not obtained by UAU1 method; Spider biofilm repressed | -2.35 | -3.3 | -3.21 |
| *C1_10710C_A* | Protein similar to S. cerevisiae Yor378w; MFS family transporter; transposon mutation affects filamentous growth; null mutants are viable; fungal-specific (no human or murine homolog) | -2.52 | -2.4 | -2.35 |
| *C1_05830W_A* | Ortholog(s) have trans-aconitate 3-methyltransferase activity and cytosol localization | -2.58 | -4.53 | -3.84 |
| TRP4 | Predicted enzyme of amino acid biosynthesis; upregulated in biofilm; regulated by Gcn2p and Gcn4p; S. cerevisiae ortholog is Gcn4p regulated | -2.59 | -2.91 | -2.15 |
| *C4_01800W_A* | Protein with a dienelactone hydrolase domain; Hap43-repressed gene | -2.59 | -2.9 | -3.18 |
| FGR41 | Putative GPI-anchored adhesin-like protein; transposon mutation affects filamentous growth; Spider biofilm repressed | -2.68 | -2.85 | -2.71 |
| MEP1 | Ammonium permease; Mep1 more efficient permease than Mep2, Mep2 has additional regulatory role; 11 predicted transmembrane regions; low mRNA abundance; hyphal downregulated; flow model biofilm induced | -2.87 | -3.76 | -2.46 |
| HOM3 | Putative L-aspartate 4-P-transferase; fungal-specific (no human or murine homolog); regulated by Gcn2 and Gcn4; early-stage flow model biofilm induced | -2.87 | -3.4 | -3.53 |
| *C6_03390W_A* | Mitochondrial dicarboxylate transporter; possibly an essential gene, disruptants not obtained by UAU1 method | -2.89 | -2.5 | -2.78 |
| MDR1 | Plasma membrane MDR/MFS multidrug efflux pump; methotrexate is preferred substrate; overexpression in drug-resistant clinical isolates confers fluconazole resistance; repressed in young biofilms; rat catheter biofilm induced | -3.12 | -3.09 | -2.47 |
| RNR22 | Putative ribonucleoside diphosphate reductase;colony morphology-related gene regulation by Ssn6; transcript regulated by tyrosol and cell density; Hap43-repressed; Spider biofilm induced | -3.32 | -2.98 | -3.71 |
