# Supplementary material for: Lrg1 Regulates β (1,3)-Glucan Masking in Candida albicans through the Cek1 MAP Kinase Pathway
Source: mBio. 2019 Sep 17;10(5):e01767-19. doi: 10.1128/mBio.01767-19 (PMC6751057; doi:10.1128/mBio.01767-19)
Supplement: TEXT S1 [file mBio.01767-19-s0001.docx]

**Text S1: Strain construction**

Strains, plasmids, and primers are listed below the text in separate tables. To generate the *LRG1* reintegrated strain, primers TCO68/TCO69 were used to amplify the *LRG1* open reading frame (ORF) and 200bp of terminator as a SacI-NotI fragment, which was cloned into the pBT1 vector between the constitutive *ENO1* promoter and the SAT1 selective marker. The resultant pTC7 was cut with MscI and the linearized fragment was transformed to the *lrg1ΔΔ* competent cells by electroporation to create the TC14 mutant. The transformation method has been described in (1).

To generate the homozygous deletion of *STE11*, the same protocol was utilized as described in (2) with minor modification. ~500 bp of 5’ promoter and 3’ terminator flanking the *STE11* ORF were amplified as KpnI-XhoI and NotI-SacI fragments, by using the primers TCO155+TCO156 and TCO157+TCO158, respectively. These two fragments were cloned to the corresponding ends of the SAT1-flipper plasmid pYLC146, respectively (3). The resulting plasmid pTC71 was digested with KpnI and SacI, and the larger fragment was transformed into *lrg1ΔΔ*. The nourseothricin-resistant transformants were further cultured in YPM medium containing maltose as a carbon source to induce *caFLP* expression, which is under *MAL2* promoter regulation. Successful deletion of one *STE11* allele was confirmed by PCR with the primers TCO159+JCO95. To delete the second *STE11* allele in the resultant *Candida* strain TC41, primers TCO162+TCO163 were used to amplify the tetracycline-repressing promoter *P_tetOFF_* with the template of plasmid pWTF1(4). The N-terminal of *P_tetOFF_* was flanked by 60bp of homology to the *STE11* promoter and the C-terminal was flanked by 60 bp of homology to the *STE11* open reading frame (ORF) located at 1402-1461 bp. The PCR product was gel-purified (Qiagen Inc., Germany), and transformed into the competent *Candida* TC41 cells, and then plated on YPD plates with 2mg/ml hygromycin B. The primers TCO181+TCO57 were utilized for colony PCR to confirm if the pWTF1 fragment replaced the N-terminal 1401bp of *STE11* ORF on the chromosome.

To generate the *Candida* strain which expresses the doxycycline regulated *P_tetOFF_-STE11^ΔN467^*, the primers TCO162+TCO163 were utilized as mentioned above to amplify the tetracycline-repressing promoter from the template of plasmid pWTF1 (4). The PCR product was transformed to the wild-type DAY286 competent cells, and then plated on the YPD plates with 2mg/ml of hygromycin B. TCO168+TCO169 were used to check if pWTF1 was integrated into the chromosome.

To generate the GST-RID-6×His construct, primers TCO139+TCO140 were used to amplify the RID domain located at 1111-1908bp of the *CaPKC1* ORF as a *BamHI-SalI* fragment by using *C. albicans* genomics DNA as template, which was then cloned into the expression vector pGEX to create pTC55. The resultant plasmid was transformed into competent *E. coli* BL21 cells for high protein expression. Primers TCO165+TCO92 were used to amplify a *Candida*-adapted hygromycin B resistance marker from pRB436 as a NotI-XbaI fragment. The PCR product was ligated to the corresponding sites in pYGS1244 (5), in which Rho1 was tagged with 6×*myc* at the N-terminus. The resultant pTC73 was transformed into the *Candida* strains of interest.

To tag the N-terminus of Rac1 with GFP, TCO170+TCO175 were used to amplify the *RAC1* ORF flanked by RsrII and Mlu from genomic DNA extracted from the *C.albicans* DAY286 strain. The PCR product was digested and inserted into the corresponding locus of the plasmid pTC33 to replace the RID domain. TRO993 was used for sequencing to check if the *RAC1* was ligated into the plasmid in the correct orientation. The resultant plasmid pTC76 was linearized by StuI restriction enzyme, followed by electroporation into *Candida* competent cells. The positive transformants selected on YPD with nourseothricin were further screened for GFP fluorescence by performing microscopy with a LEICA DM5500B epi-fluorescent microscope with a Hamamatsu Orca-ER CCD digital camera (Model#C4742-80-12AG).

To generate the hyperactive *RAS1^G13V^* allele, the primers TCO185+TCO186 were used to PCR amplify the *RAS1^G13V^* allele from the genome of the Ca79 strain (6).and introduce NotI and SacI restriction sites. The PCR product was digested and ligated into the plasmid pBT1. TCO187 was used for sequencing to check if the *RAS1^G13V^* allele was inserted with the correct orientation. The resultant plasmid pTC78 was linearized with BglII and transformed to the *C. albicans* competent cells of interest. The positive colonies were selected on YPD plates with nourseothricin and TCO185 and TCO186 were used to check if the fragment was located on the chromosome.

**References for Text S1.**

1. De Backer MD, Maes D, Vandoninck S, Logghe M, Contreras R, Luyten WH. 1999. Transformation of Candida albicans by electroporation. Yeast 15:1609-18.

2. Reuss O, Vik A, Kolter R, Morschhauser J. 2004. The SAT1 flipper, an optimized tool for gene disruption in Candida albicans. Gene 341:119-27.

3. Chen Y-L, Montedonico AE, Kauffman S, Dunlap JR, Menn F-M, Reynolds TB. 2010. Phosphatidylserine synthase and phosphatidylserine decarboxylase are essential for cell wall integrity and virulence inCandida albicans. Molecular Microbiology 75:1112-1132.

4. Lai WC, Sun HF, Lin PH, Ho Lin HL, Shieh JC. 2016. A new rapid and efficient system with dominant selection developed to inactivate and conditionally express genes in Candida albicans. Curr Genet 62:213-35.

5. Yang SL, Zeng G, Chan FY, Wang YM, Yang D, Wang Y. 2018. Sac7 and Rho1 regulate the white-to-opaque switching in Candida albicans. Sci Rep 8:875.

6. Feng QH, Summers E, Guo B, Fink G. 1999. Ras signaling is required for serum-induced hyphal differentiation in Candida albicans. Journal of Bacteriology 181:6339-6346.

***C. albicans* strains used in this study**

| Strain | Parent | Genotype | Source or reference |
| --- | --- | --- | --- |
| SC5314 | Clinical isolate | Prototrophic wild type | (1) |
| YLC337 | SC5314 | *cho1ΔΔ* | (2) |
| DAY286 | SC5314 | *ura3::imm434/ura3::imm434 iro1/iro1::imm434 his1::hisG/his1::hisG arg4/arg4* | (3) |
| *lrg1Δ/Δ* | DAY286 | *ura3::imm434/ura3::imm434 iro1/iro1::imm434 his1::hisG/his1::hisG arg4/arg4*  *lrg1::Tn7-UAU1/lrg1::Tn7-URA3* | (4) |
| TC14 | *lrg1Δ/Δ* | *lrg1ΔΔ*:: *P_ENO1_-LRG1* | This study |
| TC41 | *lrg1Δ/Δ* | *lrg1ΔΔste11Δ/STE11* | This study |
| TC48 | TC41 | *lrg1ΔΔ ste11ΔΔ::P_tetOFF_ -STE11^ΔN467^* | This study |
| TC128 | DAY286 | DAY286 with 6×myc-Rho1 | This study |
| TC131 | *lrg1ΔΔ* | *lrg1ΔΔ* with 6×myc-Rho1 | This study |
| TC132 | *lrg1ΔΔ::R* | *lrg1ΔΔ:: R* with 6×myc-Rho1 | This study |
| TC147 | DAY286 | DAY286+*P_tetOFF_ -STE11^ΔN467^* | This study |
| TC44 | DAY286 | DAY286 with GFP-Rac1 | This study |
| TC158 | *lrg1ΔΔ* | *lrg1ΔΔ* with GFP-Rac1 | This study |
| CAI-4 | SC5314 | *URA3/ura3*::imm434  *IRO1/iro1*::imm434 | (5) |
| Ca79 | CAI-4 | *RAS1/RAS1/P_MAL2_-RAS1^G13V^* | (6) |
| TC159 | DAY286 | DAY286*+P_ENO1_-RAS1^G13V^-1* | This study |
| TC160 | DAY286 | DAY286*+ P_ENO1_-RAS1^G13V^-2* | This study |

**References for *Candida* strains**

1. Gillum AM, Tsay EY, Kirsch DR. 1984. Isolation of the Candida albicans gene for orotidine-5'-phosphate decarboxylase by complementation of S. cerevisiae ura3 and E. coli pyrF mutations. Mol Gen Genet 198:179-82.

2. Chen Y-L, Montedonico AE, Kauffman S, Dunlap JR, Menn F-M, Reynolds TB. 2010. Phosphatidylserine synthase and phosphatidylserine decarboxylase are essential for cell wall integrity and virulence inCandida albicans. Molecular Microbiology 75:1112-1132.

3. Davis DA, Bruno VM, Loza L, Filler SG, Mitchell AP. 2002. Candida albicans Mds3p, a conserved regulator of pH responses and virulence identified through insertional mutagenesis. Genetics 162:1573-81.

4. Enloe B, Diamond A, Mitchell AP. 2000. A single-transformation gene function test in diploid Candida albicans. J Bacteriol 182:5730-6.

5. Fonzi WA, Irwin MY. 1993. Isogenic strain construction and gene mapping in Candida albicans. Genetics 134:717-28.

6. Feng QH, Summers E, Guo B, Fink G. 1999. Ras signaling is required for serum-induced hyphal differentiation in Candida albicans. Journal of Bacteriology 181:6339-6346.

**Plasmids used in this study**

| Strain Name | Description | Source |
| --- | --- | --- |
| pYLC146 | FLP-CaNAT, *chloraR* | (1) |
| pBT1 | *CaNAT-P_ENO1_*, *ampR* | (2) |
| pTC7 | pENO1+*CaLRG1*, *ampR* | This study |
| pExpArg-pACT1GFPRID | pExpArg-pACT1GFPRID | (3) |
| pTC33 | pExpArg-pACT1GFPRID+*NAT^R^* | (4) |
| pTC55 | pGEX+RIDHIS in *DH5α*, *ampR* | This study |
| pTC57 | pGEX+RIDHIS in *BL21*, *ampR* | This study |
| pTC71 | *pYLC146+STE11-KO, chloraR* | This study |
| pYGS1244 | TetO-Myc-RHO1-UTR*-TetR/CIP10U | (5) |
| pTC73 | pYGS1244+*HYGRO^R^* | This study |
| pTC76 | pExpArg-pACT1GFPRAC1+*NAT^R^* | This study |
| pTC78 | pENO1+*CaRAS1^G13V^*, *ampR* | This study |
| pWTF1 | *P_tetOFF_* +*HYGRO*^R^ | (6) |

**References for Plasmids**

1. Chen Y-L, Montedonico AE, Kauffman S, Dunlap JR, Menn F-M, Reynolds TB. 2010. Phosphatidylserine synthase and phosphatidylserine decarboxylase are essential for cell wall integrity and virulence inCandida albicans. Molecular Microbiology 75:1112-1132.

2. Tams RN, Cassilly CD, Anaokar S, Brewer WT, Dinsmore JT, Chen YL, Patton-Vogt J, Reynolds TB. 2019. Overproduction of Phospholipids by the Kennedy Pathway Leads to Hypervirulence in Candida albicans. Front Microbiol 10:86.

3. Corvest V, Bogliolo S, Follette P, Arkowitz RA, Bassilana M. 2013. Spatiotemporal regulation of Rho1 and Cdc42 activity duringCandida albicansfilamentous growth. Molecular Microbiology 89:626-648.

4. Chen T, Jackson JW, Tams RN, Davis SE, Sparer TE, Reynolds TB. 2019. Exposure of Candida albicans beta (1,3)-glucan is promoted by activation of the Cek1 pathway. PLoS Genet 15:e1007892.

5. Yang SL, Zeng G, Chan FY, Wang YM, Yang D, Wang Y. 2018. Sac7 and Rho1 regulate the white-to-opaque switching in Candida albicans. Sci Rep 8:875.

6. Lai WC, Sun HF, Lin PH, Ho Lin HL, Shieh JC. 2016. A new rapid and efficient system with dominant selection developed to inactivate and conditionally express genes in Candida albicans. Curr Genet 62:213-35.

Primers used in this study

| Name | Sequence | Description |
| --- | --- | --- |
| TCO57 | TTTCCACAATCAAACATCCAA | Primer-R sits at the C-terminus of *STE11* to check if *P_tetOFF_-STE11^ΔN467^* replaced the 2nd allele of *STE11* in TC41 |
| TCO69 | AAAAGCGGCCGCATGAAGCATTCGTTTGATAC | Primer-F to amplify *CaLRG* ORF and Terminator |
| TCO68 | AAAAGAGCTCGGTTTAGATTAAGCAGAGTT | Primer-R to amplify *CaLRG* ORF and Terminator |
| TCO92 | AAAGCGGCCGCTCGATATTGCTAGAGGCAAA | Primer-F to amplify Hygromycin resistance B gene with NotI flanking |
| TCO139 | AAAAGGATCCTTAGAACCAGGTGGTCAAAT | Primer-F to amplify RID domain located at 1111-1908bp of *CaPKC1* ORF flanking with BamHI |
| TCO140 | AAAAGTCGACCCGACGTCTAGTAGAAGGA | Primer-R to amplify RID domain located at 1111-1908bp of *CaPKC1* ORF flanking with SalI |
| TCO155 | AAAAGGTACCGATGAACAGGTATTAGTATCA | Primer-F to amplify 500bp of CaSTE11 promoter with KpnI flanking |
| TCO156 | AAAACTCGAGGACTGTTTGTTAGATTTGAGT | Primer-R to amplify 500bp of CaSTE11 promoter with XhoI flanking |
| TCO157 | AAAAGCGGCCGCCCATTAATTATGTCGAAACAA | Primer-F to amplify 500bp of CaSTE11 terminator with NotI flanking |
| TCO158 | AAAAGAGCTCCAAGTAGGTTCACTGTAGAT | Primer-R to amplify 500bp of CaSTE11 terminator with SacI flanking |
| TCO159 | AGGAGAGATTAAAACTTGGA | Primer-F to check if the pYLC146+STE11 KO construct was integrated into chromosome |
| TCO162 | ATATAGAAAACCTAATACATTAGTTCGTGTGTATATGGTACTCAAATCTAACAAACAGTCCTTGGACTCTTGAATCCGCGG | Primer -F to amplify tetracycline-repressing promoter flanking by 60-mer homology of *STE11* promoter |
| TCO163 | AATCCTATGCAATGCAGTTCCCCCACTTAATGATTTTGACGATATTATGGATCTCGAATCCATGACTGCACCAGCTCCGGTACCACT | Primer -R to amplify tetracycline-repressing promoter flanking by 60-mer homology of STE11 ORF located at 1402-1461bp |
| TCO165 | AAATCTAGAATTTTATGATGGAATGAATGG | Primer-R to amplify Hygronyocin B resistance gene with XbaI flanking |
| TCO168 | CTGTTCTTTCATGGTATTCT | Primer-F sits at -117bp of the *STE11* promoter to check if *P_tetOFF_-STE11^ΔN467^* was integrated into the chromosome in the CaTC147 mutant |
| TCO169 | ACTGATTTATTCAATTGATGG | Primer-R sits within the pTDH3 of pWTF1, and is to check if *P_tetOFF_-STE11^ΔN467^* construct was integrated into the chromosome in the CaTC147 mutant |
| TCO170 | AAAACGGACCGTGTACCCATACGATGTCCCAGACTACGCAATGAGAAGCATTAAATCAGTCGTAG | Primer-F to amplify *RAC1* ORF with RsrII flanking |
| TCO175 | AAAAACGCGTCCCATGATTATAATATAGTACATTTTTTAGCTCTC | Primer-R to amplify *RAC1* ORF with MluI flanking |
| TCO181 | CTACTCGATTGAGTAATAGA | Primer-F at the miniOP4 promoter of pWTF1 to check if *P_tetOFF_-STE11^ΔN467^* is replaced the 2nd allele of *STE11* in TC41 |
| TCO185 | AAAAGCGGCCGCATAGACGAAGACACGGTATA | Primer-F to amplify *RAS1^G13V^* with NotI flanking |
| TCO186 | AAAAGAGCTCCTCCAGAAAGATCATTTGGT | Primer-R to amplify *RAS1^G13V^* with SacI flanking |
| TCO187 | AACGTAACTTGGAAACAATC | Primer used to sequence if the *P_ENO1_-RAS1^G13V^* construct was inserted with correct orientation in the plasmid pTC78. |
| TRO993 | TTGGTGATGGTCCAGTCTTGT | Primer-F at the EGFP region of pTC76 to check if EGFP and RAC1 were ligated in a correct orientation |
| JCO95 | GATTATTAGTTAAACCACTGC | CaSAT1’s MAL2p reverse checking primer |
